# Effect of inactivated nature‐derived microbial composition on mouse immune system

**DOI:** 10.1002/iid3.579

**Published:** 2021-12-06

**Authors:** Martín Ignacio González‐Rodríguez, Noora Nurminen, Laura Kummola, Olli H. Laitinen, Sami Oikarinen, Anirudra Parajuli, Tanja Salomaa, Iida Mäkelä, Marja I. Roslund, Aki Sinkkonen, Heikki Hyöty, Ilkka S. Junttila, for the ADELE Research Group

**Affiliations:** ^1^ Faculty of Medicine and Health Technology Tampere University Tampere Finland; ^2^ Department of Clinical Microbiology Fimlab Laboratories Finland; ^3^ Department of Medicine, Karolinska Institutet Center for infectious medicine (CIM) Huddinge Sweden; ^4^ Ecosystems and Environment Research Programme University of Helsinki Helsinki Finland; ^5^ Department of Garden Technologies, Horticulture Technologies Natural Resources Institute Finland Finland

**Keywords:** rodent, animals, autoimmunity, diseases, T cells, cells

## Abstract

**Introduction:**

The hygiene hypothesis suggests that decrease in early life infections due to increased societal‐level hygiene standards subjects one to allergic and autoimmune diseases. In this report, we have studied the effect of sterilized forest soil and plant‐based material on mouse immune system and gut microbiome.

**Methods:**

Inbred C57Bl/6 mice maintained in normal sterile environment were subjected to autoclaved forest soil‐derived powder in their bedding for 1 h a day for 3 weeks. Immune response was measured by immune cell flow cytometry, serum cytokine enzyme‐linked immunoassay (ELISA) and quantitative polymerase chain reaction (qPCR) analysis. Furthermore, the mouse gut microbiome was analyzed by sequencing.

**Results:**

When compared to control mice, mice treated with soil‐derived powder had decreased level of pro‐inflammatory cytokines namely interleukin (IL)−17F and IL‐21 in the serum. Furthermore, splenocytes from mice treated with soil‐derived powder expressed less IL‐1b, IL‐5, IL‐6, IL‐13, and tumor necrosis factor (TNF) upon cell activation. Gut microbiome appeared to be stabilized by the treatment.

**Conclusions:**

These results provide insights on the effect of biodiversity on murine immune system in sterile environment. Subjecting mice to soil‐based plant and microbe structures appears to elicit immune response that could be beneficial, for example, in type 2 inflammation‐related diseases, that is, allergic diseases.

## Graphical abstract

Autoclaved forest soil extract was added to the bedding of standard laboratory mice. The immune response of mice was measured indicating decreased pro‐inflammatory cytokine production in soil‐subjected mice.

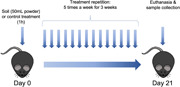

## 1 IMPORTANCE

Biodiversity and changes in biodiversity may be important factors in regulating immune system activation. We study here, how the immune system of sterile laboratory animals is affected by exposure to autoclaved antigens from boreal forest soil, containing environmental plant, yeast, bacteria, and virus particles. We found that soil‐treated mice showed decreased pro‐inflammatory cytokine expression, both in soil‐subjected mice and in in vitro activated splenocytes from soil‐subjected mice. In particular, the decreased expression of allergic inflammation‐related cytokines in response to splenocyte activation from soil‐treated mice is of interest; this would imply that nonpathogenic microbial encounters would generate an antiallergic environment. In total, these results offer one explanation, why biodiversity is important in regulating tolerance of the immune system.

## 2 INTRODUCTION

Epidemiological studies have associated the epidemic of immune‐mediated disorders, such as allergies and autoimmune diseases, with the adoption of a modern, urban lifestyle.^1^, ^2^ Indeed, the prevalence of atopic dermatitis in industrialized countries in the past decades has increased up to 30% in children and 10% in adults.^3^ Additionally, the incidence of many autoimmune diseases, such as type 1 diabetes (T1D), celiac disease, and inflammatory bowel diseases (IBDs), has increased concomitantly.^4^, ^5^ Hygiene hypothesis suggests that high sanitation standards and the resulting decrease in infectious burden may lead to aberrant immune reactions against normally harmless antigens such as pollen or animal dandruff.^2^, ^6^, ^7^, ^8^

The biodiversity hypothesis has further expanded the hygiene hypothesis, underlining the importance of environmental biodiversity and its impact on the gut and skin microbiota in the development of a healthy immune system.^9^, ^10^ Ample evidence supports this; rural environment, exposure to farm and household animals,^11^ older siblings and a diet rich in fiber are all known to be associated with a diverse microbiome and a reduced risk of immune dysfunction.^12^, ^13^, ^14^, ^15^, ^16^ We have recently shown that living in an agricultural environment during the first year of life decreased the risk of T1D.^17^

Therapeutic interventions that aim to provide immunomodulatory, “protective” microbiota have been under intense investigation lately. Most studies have investigated the effect of probiotics, containing only one or few strains of bacteria in the prevention or treatment of conditions varying from allergy to obesity and cancer.^18^, ^19^, ^20^, ^21^, ^22^ In addition, recent studies have introduced a concept, in which exposure to natural, highly diverse soil bacteria could have immunomodulatory effects. It was shown that exposing children living in urban environments to microbiologically diverse soil and vegetation increased the diversity of their microbiome, which was associated with the expression of cytokines such as transforming growth factor‐beta (TGF‐β), the increased interleukin (IL)‐10/IL‐17A ratio and an increase in regulatory T cells in peripheral blood.^23^ Another trial showed that rubbing similar soil‐based material on hands daily, for 2 weeks, modulated the stool microbiome. The change in bacterial diversity was connected with the level of TGF‐β expression in blood peripheral mononuclear cells.^24^

Inbred mouse strains living in a clean environment are traditionally used for immunological studies. The sterile environment of animal facilities greatly helps in the evaluation of the function of the immune system and its genetic regulation, but the exclusion of external microbes that are present in the natural mouse environment may also modulate the immune response. Here, we have studied the effect of autoclaved soil and plant‐based material (aka soil powder) on the immune system of C57Bl/6 mice. We prepared an organic powder with high microbiological diversity from microbially diverse organic soil and plant materials. We then subjected mice externally to the autoclaved powder and measured subsequently the immune response in these mice. Interestingly, we found a reduced expression of pro‐inflammatory cytokines, IL‐17F, and IL‐21, in the serum of mice treated with the soil powder. CD4 + T cells from soil‐treated mice showed decreased expression of CD69 activation marker upon stimulation, as well as decreased expression of IL‐1β, IL‐5, IL‐6, IL‐13, and tumor necrosis factor (TNF). Overall, autoclaved soil powder might provide an additional tool to provide safe immunoregulation by down‐tuning harmful immune responses.

## 3 RESULTS

### 3.1 Subjecting mice to boreal forest soil powder had no adverse effects on mice's health status

**Figure 1:**
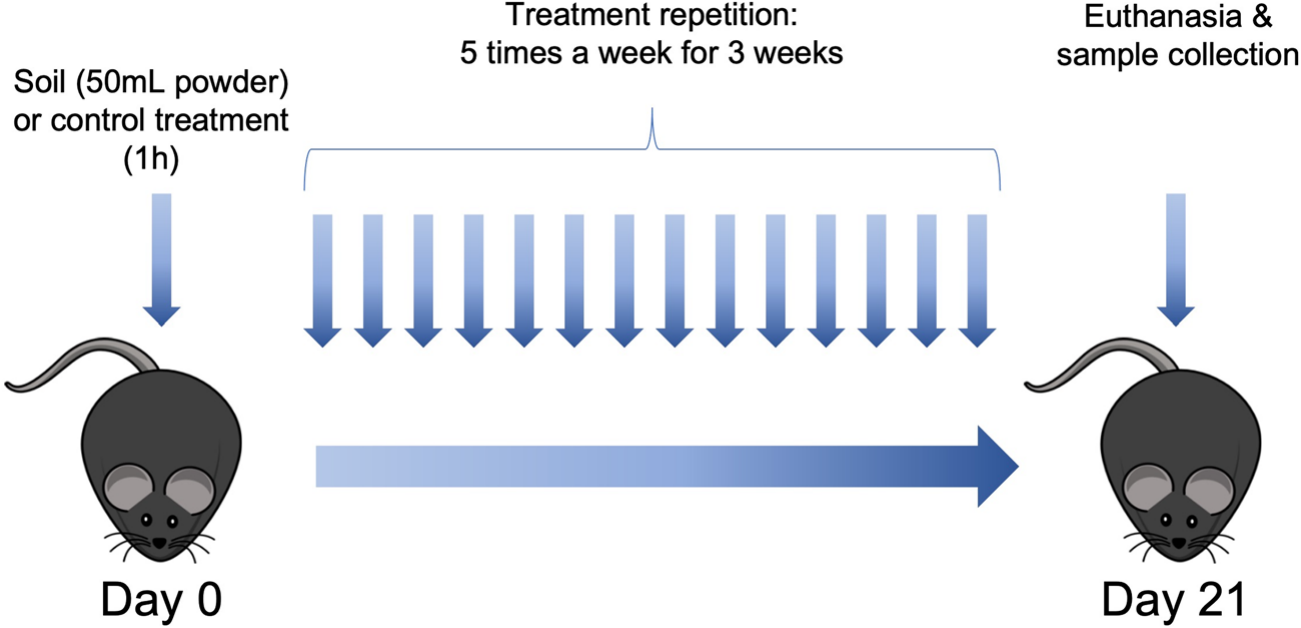
Experimental setup for soil extract exposure of mice. The soil exposure was performed in 6–7‐week‐old Female mice (C57BL/6) for 1 h each day to normal mouse bedding alone (control group) or bedding mixed with 50 ml of soil powder and then returned to their normal cage. Soil dose was measured using 50 mlSchott glass bottles (Kisker Biotech GmbH & Co. KG). This procedure was repeated five times a week for 3 weeks. Identical experiment was performed twice to obtain a total of eight mice per treatment group. At Day 21 the animals were euthanized, and organ samples were collected

Details of the production of the soil extract are provided in Section 5. Briefly, composted crushed tree bark and mulch, dung, deciduous leaf litter, peat and agricultural sludge, and dried and crushed Sphagnum moss were crushed and mixed thoroughly, diluted to water, freeze‐dried and autoclaved. Microbial communities of this soil‐derived powder are shown in Table S1. To study how the soil powder affects the murine immune system, we subjected mice (*n* = 8) to the powder while leaving the control group (*n* = 8) with normal bedding. We added the soil powder to the bedding of mice for one hour, 5 days in a row, for 3 weeks (Figure 1). We then euthanized the mice, harvested organs and serum for subsequent analyses. During the 3‐week exposure period, no animal welfare problems, change in behavior, loss of weight nor any other signs of harm were observed, indicating that the soil powder was safe to use for the animals.

### 3.2 Cytokine expression in mice was shaped by the soil powder exposure

**Figure 2:**
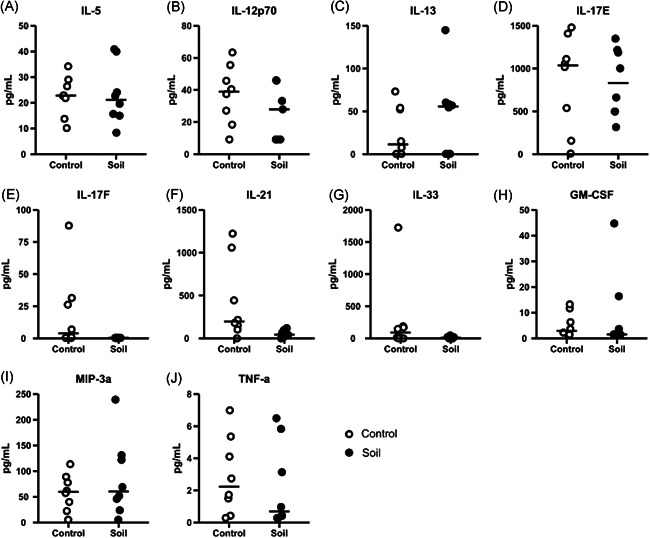
Soil exposure downregulate the levels of interleukin (IL)‐17F and IL‐21 in mice serums. Serums were collected after euthanasia (Day 21) and later analyzed using Milliplex Mouse Th17 kit. White open circles corresponded to control animals and closed black circles to soil‐exposed animals. Following cytokines were measured: (A) IL‐5, (B) IL‐12p70, (C) IL‐13, (D) IL‐17E, (E) IL‐17F, (F) IL‐21, (G) IL‐33, (H) granulocyte‐macrophage colony‐stimulating factor (GM‐CSF), (I) macrophage inflammatory protein‐3 alpha (MIP‐3a), and (J) tumor necrosis factor (TNF). Data are shown as scatter plots with mean as a bar (*n* = 8 per group). In graphs, each symbol represents an individual mouse, lines indicate the median. The data was analyzed with Mann–Whitney *U* test, *p < .05, **p < .01, ***p < .001

Previous reports indicated that exposure to powder changed the cytokine profile in the human sera.^24^ Initially, to understand whether the exposure of mouse to inactivated soil powder had any effects on the serum cytokine concentrations, a set of cytokines was studied. A significant reduction was found in the serum concentration of the Th17‐related cytokines IL‐17F and IL‐21 in mice exposed to the soil mixture compared to the control animals (Figure 2E,F). The mean cytokine concentrations for the exposed mice and control mice were 19.2 and 0.33 pg/ml for IL‐17F (*p* = .01), and 422.0 pg/ml and 53.1 pg/ml for IL‐21 (*p* = .02), respectively. Meanwhile no differences were found in other lymphocyte‐related cytokines such as IL‐5 and IL‐13 (Figure 2A,C) or innate immune‐related cytokines such as IL‐12p70, IL‐17E, IL‐33, GM‐CSF, macrophage inflammatory protein‐3 alpha (MIP‐3a), and TNF (Figure 2B,D,G–J).

### 3.3 Effect of soil powder on various T cell compartments

**Figure 3:**
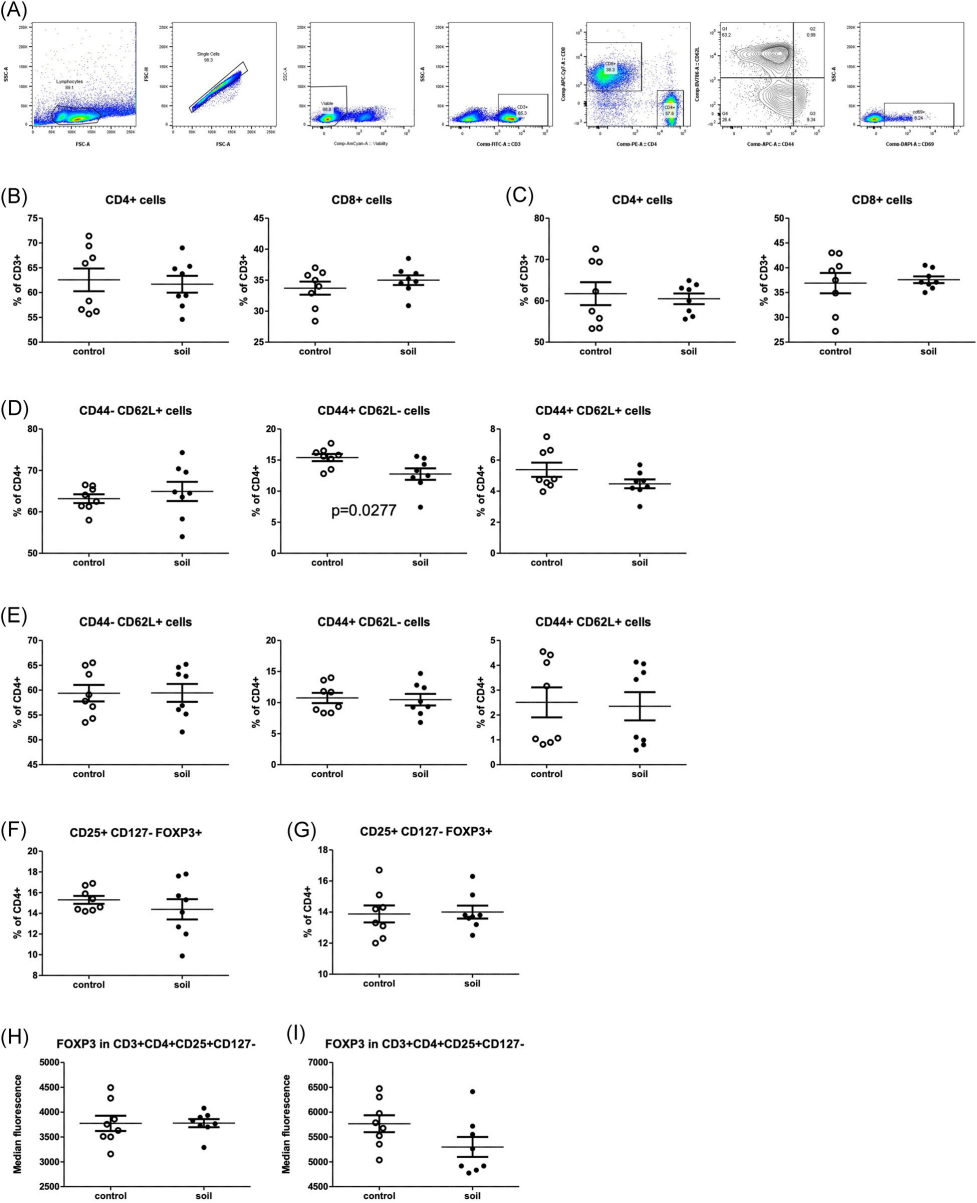
Effect of soil exposure on T cell memory and T regs in spleen and mesenteric lymph nodes (mLN). On Day 21, single‐cell suspension were prepared from spleen and mLNs. Cells were analyzed using flow cytometry, open circles represent control mice and closed circles soil treated animals. (A) Representative chart of flow cytometry gating strategy to identify different T cell populations. CD4^+^ and CD8^+^ T cells were assessed as percentage of total CD3^+^ cells both in (B) spleen and (C) mLN. Naïve (CD44^‐^/CD62L^+^), effector memory (CD44^+^/CD62L^−^) and central memory (CD44^+^/CD62L^+^) T cells were quantified and represented as proportion of total CD4^+^ in (D) spleen and (E) mLN. Regulatory T cells (CD25^+^/CD127^−^/FOXP3^+^) were measured and represented as percentage of CD4^+^ cells in (F) spleen and (G) mLN. Finally, FoxP3 geometric mean of fluorescent intensity (GMFI) was measured in regulatory T cells from (H) spleen and (I) mLN. Values are shown as percents with median and SEM is indicated (*n* = 8 mice per group)

Many regulatory cytokines in the immune system are expressed by T lymphocytes. Assessment of T cells in secondary lymphoid organs such as spleen and mesenteric lymph nodes (mLNs) was performed using flow cytometry (Figure 3A and Table 1). No significant differences in the proportion of the CD4 and CD8 T cells from soil‐treated animals compared to the control group in spleen nor in mLNs were observed (Figure 3B,C).

**Table 1:** T cell memory and Treg phenoptypes in soil‐treated and control groups

Organ	Surface markers		T cell group	*p* value	Organ	Surface markers		T cell group	*p* value
Spleen	CD8^+^		Cytotoxic T cells	.3405 (ns)	mLN	CD8^+^		Cytotoxic T cells	.7558 (ns)
	CD4^+^		Helper T cells	.7642 (ns)		CD4^+^		Helper T cells	.6926 (ns)
	CD25^+^ CD127^−^		Tregs	.3051 (ns)		CD25^+^ CD127^−^		Tregs	.5781 (ns)
	FOXP3 (MFI)		Tregs	.9826 (ns)		FOXP3 (MFI)		Tregs	.0964 (ns)
	CD4^+^	CD44^−^ CD62L^+^	Naive	.5045 (ns)		CD4^+^	CD44^−^ CD62L^+^	Naive	.9800 (ns)
		CD44^+^ CD62L^+^	Central memory	.1135 (ns)			CD44^+^ CD62L^+^	Central memory	.8520 (ns)
		CD44^+^ CD62L^−^	Effector memory	.0277			CD44^+^ CD62L^−^	Effector memory	.8231 (ns)
	CD8^+^	CD44^−^ CD62L^+^	Naive	.7351 (ns)		CD8^+^	CD44^−^ CD62L^+^	Naive	.3251 (ns)
		CD44^+^ CD62L^+^	Central memory	.2398 (ns)			CD44^+^ CD62L^+^	Central memory	.9615 (ns)
		CD44^+^ CD62L^−^	Effector memory	.9785 (ns)			CD44^+^ CD62L^−^	Effector memory	.4250 (ns)

*Note*: Mice were either treated with soil (*n* = 8) or left untreated for control (*n* = 8) as indicated in Figure 1. On Day 21, spleen and lymph nodes from soil‐treated and control mice were stained for T cell memory and T reg markers. Data were analyzed using unpaired, two‐tailed *t* test. *p* < .05 indicates significant differences between groups.

We also studied the proportion of CD44^‐^CD62L^+^ naïve (NA), CD44^+^CD62L^−^ effector memory (EM) and CD44^+^CD62L^+^ central memory (CM) cells in CD4 and CD8 T cells (Figure 3D,E). No difference was observed in absolute number or in the proportion of NA and CM T cells in the spleen (Figure 3D). However, the proportion of EM T cells was decreased in the spleens of soil‐exposed animals compared to the control animals (*p* = .03, Figure 3D middle panel). In mLN, no differences were found in the absolute numbers or relative proportions of NA, EF, or CM T cells between the exposure and control groups (Figure 3E), and the activation status of these cells (CD69 expression) was similar in both groups (Figure S1).

Since regulatory T cells have been found to increase in peripheral blood of humans subjected to biodiverse soil and plant materials,^23^ we next analyzed the proportion of regulatory T cells (Tregs) in both spleen and lymph nodes. No difference was found in the overall proportion of Tregs within the CD4^+^ T cell compartment in the spleen or in lymph nodes (Figure 3F,G). Finally, FOXP3 expression in the spleen and mLN CD3^+^CD25^+^CD127^‐^ Tregs was not affected in the animals treated with soil when compared with the control group (Figure 3H,I).

### 3.4 Differential immune‐related gene expression during soil powder exposure

**Figure 4:**
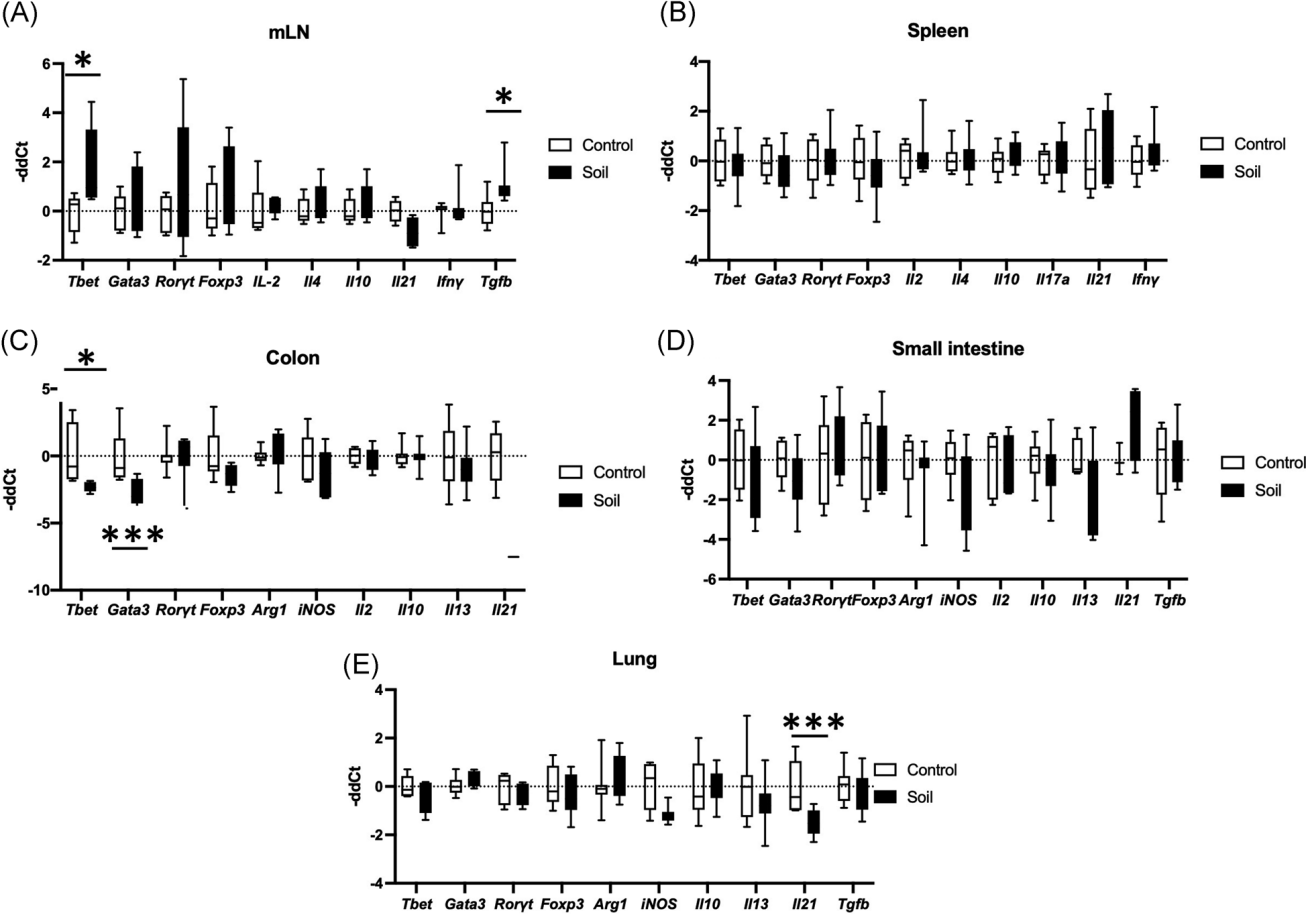
Gene expression changes in different organs after soil exposure. Mice were either treated with soil (*n* = 8) or left untreated (*n* = 8) as in Figure 1. RNA was then extracted from lymphoid organs (spleen and mesenteric lymph node [mLN]), intestine (small intestine and colon), and lung. Using quantitative polymerase chain reaction (qPCR) gene expression was measured. Open bars represent control animals and close black bars soil treated animals. T cell lineage‐associated genes (Tbet, Gata3, Rorɣt, and Foxp3), and cytokine expression (IL‐2, IL‐4, IL‐10, IL‐17a, IL‐21, and IFN‐ɣ) were measured in (A) mLN (Tgfβ was also analyzed for mLN) and (B) spleen. For the gastrointestinal organs, T cell lineage associated genes (Tbet, Gata3, Rorɣt, and Foxp3), macrophage activation associated genes (Arg1 and iNos) and cytokine expression (IL‐2, IL‐10, IL‐13, and IL‐21) were measured in (C) colon and (D) small intestine (Tgfβ was also analyzed for small intestine). Finally, T cell lineage associated genes (Tbet, Gata3, Rorɣt, and Foxp3), macrophage activation associated genes (Arg1 and iNos), and cytokine expression (IL‐10, IL‐13, IL‐21, and Tgfβ) were measured in (E) lung. Values are shown as box and whiskers (25–75 percentile). The data were analyzed with Mann–Whitney *U* test, *p < .05, **p < .01, ***p < .001

To gain further insights into the effects of soil powder exposure on the immune system on the tissue level, we measured inflammation‐related gene expression by reverse transcription‐quantitative polymerase chain reaction (RT‐qPCR) in secondary lymphoid organs, and organs directly exposed to the soil powder via ingestion or inhalation (i.e., lung and gut). In the mLN *Tbet* (Th1‐related transcription factor) was upregulated in the soil‐treated animals (*p* = .03; Figure 4A). Furthermore, *Tgfβ* levels were downregulated in mLN from soil‐treated animals compared to the control group. No differences were seen in other cytokine gene expression or in Th2‐ and Th17‐related transcription factors *Gata3* and *Rorgt*, between the groups (Figure 4A). In spleen, the expression of transcription factors or T‐cell phenotype‐associated cytokines (*Tbet, Gata3, Rorgt* and *Foxp3, Ifnɣ, Il17A, Il4, Il10*, and *Il21*) did not differ between the intervention and control groups (Figure 4B).

Next, we analyzed immune‐associated genes in organs directly exposed to the soil powder such as the small intestine, colon, and lung. When the same set of genes was analyzed in colon no difference was found in the cytokine expression but both *Tbet* and *Gata3* were downregulated in soil powder‐exposed animals compared to the control group (*p* = .0159 and *p* = .0019, Figure 4C). When cytokine and T‐cell phenotype‐associated genes were analyzed in small intestine, no difference was found between intervention and control groups (Figure 4D). Finally, in lung tissue, the genes related with T cell phenotype such as *Tbet, Gata3*, and *Rorgt* were not altered. However, *Il21* expression was downregulated in lung tissue of the soil‐treated animals compared to the control group (*p* = .0037), while *Il10, Il13*, an*d Tgfβ* remained unchanged (Figure 4E).

### 3.5 CD4^+^ T cell activation and cytokine production are reduced by soil powder treatment

**Figure 5:**
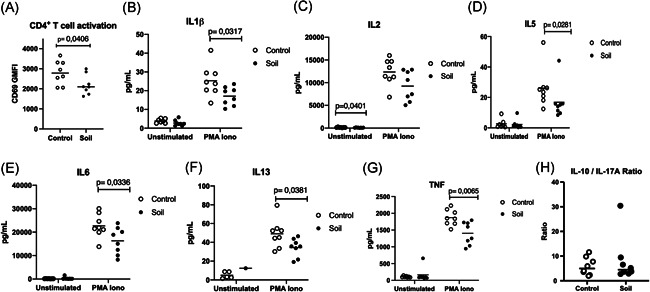
CD4^+^ T cell activation and cytokine production is reduced by soil exposure. Mice were either treated with soil (*n* = 8) or left untreated (*n* = 8) as indicated in Figure 1. On Day 21, single‐cell suspension from splenocytes was prepared, cells were rested overnight and then either left unstimulated or stimulated with phorbol 12‐myristate 13‐acetate (PMA)/Ionomycin for 4 h and splenocytes harvested. CD4+T cell activation was measured by flow cytometry and (A) CD69 geometric mean fluorescence intensity (gMFI) was quantified (*t* test) open circles represent control animals and closed circles soil exposed mice. Supernatants were collected 24 h poststimulation (B) interleukin (IL)‐1β (*t* test), (C) IL‐2 (MW test), (D) IL‐5 (MW test), (E) IL‐6 (*t *test), (F) IL‐13 (*t* test), and (G) tumor necrosis factor (TNF) (*t* test) cytokine levels were quantified using MSD mesoscale platform. IL‐10/IL17A ratio (H) from the same samples was obtained by analyzing the supernatants with Milliplex Mouse Th17 kit. Values are shown scattered dots with median, and data were analyzed using GraphPad prism (8.0). *t* test analysis was performed for parametric data and Mann–Whitney *U* test (MW) for nonparametric ones. Normal distribution was assessed using Anderson–Darling test

We next evaluated the T cell response to activation stimuli by PMA/Ionomycin between the treatment groups. We harvested splenocytes from control group and soil‐treated animals and let them rest for 16 h. The cells were then either stimulated for 4 h with PMA/Ionomycin or left unstimulated and then analyzed by flow cytometry (Figure S2A). Interestingly, the geometric mean fluorescence intensity (gMFI) of CD69 expression was decreased on CD4^+^ T cells in the soil powder treated animals when compared to the control group (*p* = .0406, Figure 5A), while the proportion of CD69^+^ activated T cells was similar (Figure S2C,D). This would suggest that while the number of activated cells was similar between the treatment groups, the activation level of individual T cell in the soil‐treated group would be decreased.

For this, we also evaluated whether the change in CD69‐reflected cell activation was associated with any type of cytokine pattern in the leukocyte culture medium. Therefore, we collected culture medium at 24‐h post‐PMA/Ionomycin stimulation and measured the expression of several cytokines in the supernatant. Interestingly, the expression of several cytokines was lower in splenocytes from soil‐treated animals. These included TNF, IL‐1β, IL‐5, IL‐6 and IL‐13 (*p* = .0065, *p* = .0317, *p* = .0281, *p* = .0336 and *p* = .0381, respectively; Figure 5B–F). Of these, IL‐5 and IL‐13 are hallmark cytokines of type 2 inflammation, involved in eosinophilia, smooth muscle cell contraction and mucus secretion, and TNF is important in several inflammatory diseases. Remarkably the production of IL‐2 was found to be significantly reduced in the unstimulated splenocytes from soil‐treated mice (*p* = .0401, Figure 5C). Previously a higher IL‐10/IL‐17A ratio in soil‐exposed children was reported.^23^ As we evaluated levels of these cytokines in the supernatants of PMA/ionomycin‐activated mouse splenocytes, no difference was found among groups (Figure 5H). Other cytokines such as IL12p70, IL‐17A, IL‐17F, IL‐25, IL33, and IFN‐ɣ were not significantly affected (Figure S3A–G).

### 3.6 Changes in the gut microbiota during the soil powder treatment

**Figure 6:**
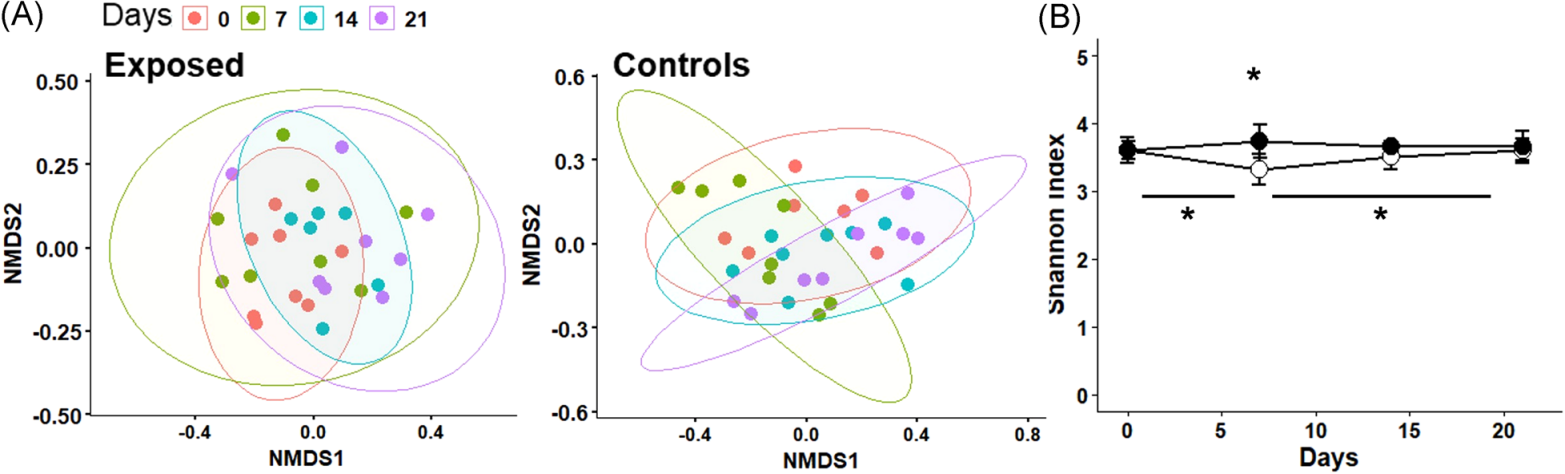
Changes in the gut microbiota during the follow‐up. Stool samples were collected weekly during the follow‐up from exposed and control mice. (A) Gut bacterial communities of the exposed (left) and control (right) mice during follow‐up (nonmetric multidimensional scaling (NMDS) of Bray–Curtis distances). (B) Shannon index measuring bacterial diversity during the follow‐up of the two study groups (open circles represent control mice and black circles represent exposed mice). Statistically significant differences marked with asterisk (*p* < .05)

Stool samples were collected on days 0, 7, 14, and 21 during the soil powder exposure and bacterial composition was measured as described in Section 5. Gut bacterial community composition shifted during the exposure period (PERMANOVA Day 0 vs. Day 21, *p* = .005) (Figure 6A left panel, Table S2). However, a stronger composition shift was seen in the control mice, in which all time points differed from day 0 (Figure 6A, right panel; Table S2). The gut bacterial composition also differed between exposed and control mice in the middle of the exposure period (PERMANOVA Day 7 *p* = .03, day 14 *p* = .02, Table S2). Compositional changes seen in the control mice were linked to the gut microbial Shannon diversity. Diversity was higher in the exposed mice compared to controls on Day 7 (*p* = .003) and the difference was linked to an early drop of diversity in control mice, which later leveled off (control mice: Day 0 vs. Day 7, *p* = .02; Day 7 vs. Day 21 *p* = .01) (Figure 6B). The abundance of phylum *Actinobacteria* decreased in both groups during the study (Table S3). The abundance of phylum *Proteobacteria* was higher in the exposed mice after a week of exposure (Day 7 adjusted *p* = .02) compared to control mice but the difference leveled off later due to a decrease of abundance in the exposed mice (Day 7 vs. Day 21, adjusted *p* = .04). Similarly, the abundance phylum *Deferribacteres* was higher in the exposed mice after a week of exposure (adjusted *p* = .01) but later the difference leveled out (Table S3). All in all, more dramatic changes were seen in the gut microbiota of the control mice compared to the mice exposed to the microbially inactivated material.

## 4 DISCUSSION

The activation of immune system is regulated by environmental factors. Down‐tuning the immune response could be beneficial in conditions where overactivated immune system can cause tissue damage and trigger and/or accelerate autoimmune or other immune‐mediated diseases. In developed societies, urbanization has been suggested to be linked to increase in allergies and autoimmune diseases.^25^ However, the link between urbanization and allergic‐/autoinflammatory diseases is likely quite complex. Quite recently, urbanization in sub‐Saharan Africa was shown to be related to a pro‐inflammatory (IFN‐γ) direction, possibly due to dietary changes in living environment.^26^ Thus, the concept of increased allergic‐/autoimmune diseases in city dwellers may be true in developed countries, but in developing countries, other factors, such as dietary changes may overdrive the effect of environmental microbiome changes.

Though the relationship between urban lifestyle and allergies is likely a complex phenomenon, any low‐cost, safe, and efficient immunomodulatory tools would be of value in clinical trials aiming to identify new treatment and prevention modalities. As there is clear evidence that, for example, touching biodiverse soil increases skin microbiota,^27^, ^28^ and farm environment in childhood has a protective role in the development of allergic diseases and type 1 diabetes,^12^, ^16^, ^17^, ^29^ approaches providing exposure to diverse natural microbial communities in urban settings might be beneficial.^30^ The beneficial effects of the exposure are believed to be mediated by a wide stimulation of several different types of pattern recognition receptors leading to downstream activation of immunological pathways. Since the recognized patterns are microbial biomolecules such as nucleic acids, lipopolysaccharide, and mannans, the material should preserve its immunomodulatory effects even when it has undergone heavy gamma radiation. To understand the relationship of external microbial exposure with the immune system, we subjected mice in sterile environment to autoclaved soil powder. Such powder is relatively easy and cost‐effective to prepare with little batch‐to‐batch variation.^23^, ^24^, ^28^

Subjecting mice to the autoclaved material in sterile environment has important difference to our previous study of nonautoclaved soil powder on humans.^23^, ^24^, ^28^ Effectively, we activated here the immune system via recognition of inactivated microbes in combination of huge diversity of microbial components that can stimulate various kinds of pattern recognition receptors. Interestingly, the number of regulatory T cells, as a read‐out of increased peripheral tolerance, was not changed in response to soil powder (Figure 3). In fact, we observed slightly decreased production of IL‐2 in splenocyte cultures from soil‐treated mice (Figure 5). The functional role of this decrease in IL‐2 production, for example, on Treg development, is difficult to assess as IL‐2 is not only important for Treg development but also for the proliferation of activated natural killer (NK) cells, T cells, and ILC2's.^31^ We also found higher expression of *Tbet* in mLN from soil‐exposed animals meanwhile its expression was reduced in colon (Figure 4). *Tbet* is a critical transcription factor involved in immune cell differentiation.^32^ It is the key regulator of Th1 cell differentiation and also suppresses Th17 cell development. Thus, the induction of *Tbet* expression might indicate higher Th1 T cells development at the intestinal lymphoid tissue.^33^, ^34^ The fact that *Tbet* expression was increased in the mLN but not in the intestinal tissue (colon or small intestine) could be attributed trafficking of T cells that have been activated by the ingested extract in the gut into the draining lymph node (mLN).^32^, ^34^, ^35^ The process of antigen presentation in the mLN would then lead to clonal expansion and enrichment of these cells into the mLN. Besides T cells, other immune cells also express Tbet such as ILCs, NK cells, and even some specific B cells.^35^, ^36^, ^37^

Importantly, we found that PMA/Ionomycin‐activated splenocytes from mice that had been treated with soil powder showed a significantly decreased expression of TNF, IL‐1β, IL‐5, IL‐6, and IL‐13. Soluble TNF selectively binds TNFR1, which induces pro‐inflammatory signals and acts as a potent neutrophil chemoattractant.^38^, ^39^ Similarly, IL‐1β and IL‐6 are pro‐inflammatory cytokines released by macrophages and dendritic cells and their upregulation have been associated with allergic reactions such as asthma and urticaria.^40^, ^41^ Furthermore, IL‐5 and IL‐13 are important CD4 T‐cells and ILC2‐derived cytokines that are involved in eosinophilia, mucus production, and airway hyperreactivity, and they have been suggested as biomarkers and therapeutic targets for type‐2 inflammation‐related disorders such as allergic asthma.^42^, ^43^, ^44^, ^45^

An interesting observation is that mice treated with the microbial powder had a lower abundance of phylum *Actinobacteria* in the gut after the whole exposure period. In addition, the treatment balanced the fluctuations in the microbial abundances and diversity during the treatment period as such fluctuations were more pronounced in the control mice (phyla *Proteobacteria* and *Deferribacteres*). Since the exposure material was microbially inactivated the changes in the gut microbial compositions were expected to be subtle. In principle, the bacterial particles in the material could serve as a source of energy for other bacteria which then could be enriched in the gut. However, no such enrichment was observed. Thus, in this experimental setting, the bacterial PRR ligands acting as immunostimulants seem to be the major drivers of the observed immunological changes, not the living bacterial component of the gut microbiota.

We also detected reduced serum expression of IL‐17F and IL‐21 (Figure 2). IL‐21 is a common gamma chain cytokine that plays a role in follicular T cell development^46^ and B cell immunoglobulin‐production.^47^ Furthermore, IL‐21 has a role in induction of CD4 T cell for producing IL‐17.^48^ The decrease of IL‐21 is particularly interesting in this context as it can regulate allergic inflammation via IgE production.^49^ IL‐21 induces inhibitor of differentiation 2 and leads to complete abrogation of anaphylaxis in mice,^50^ but also plays a role in development of autoimmune diseases such as type 1 diabetes.^49^ The mechanisms behind the reduced levels of IL‐21 and IL‐17F induced by the soil mixture remains to be elucidated, but reduced IL‐17 serum levels have been detected in children during exposure to *Lactobacillus plantarum*.^51^ Moreover, our previous studies showed increased IL‐10/IL‐17A ratio in children exposed to soil‐derived extract.^23^ However, we were not able to detect IL‐10 or IL‐17A in mouse serums. In the analysis of culture medium of the IL‐10/IL‐17 ratio did not differ between the treatment and control groups. Soil‐based intervention in daycare children showed that increased levels of TGF‐β were positively associated with Gammaproteobacteria.^23^ In the present study, no detectable levels of TGF‐β were found in mouse serum. Nevertheless, we did find elevated *Tgf‐β* expression in mLN of soil‐treated animals (Figure 4). TGF‐β has an important role in downregulation of MHC‐II expression in monocytes/macrophages and pro‐inflammatory cytokine production.^52^, ^53^ TGF‐β also inhibits T and B cell proliferation as well as the production of IL‐2, IFN‐γ, and TNF.^54^, ^55^ Furthermore, TGF‐β has been described as a critical regulator of intestinal immunity as it suppresses inflammatory responses to commensal bacteria and as impaired TGF‐β production associated with IBD susceptibility.^56^ Future experiments on how efficient such treatments might be in delaying or inhibiting experimental allergy or type 1 diabetes based on our results are warranted.

In conclusion, we demonstrated that exposing mice to inactivated biodiversity powder has potentially beneficial effects on mouse's immune system and in particular it downregulated inflammatory cytokines IL‐17F and IL‐21. In addition, CD4 + T cells from mice exposed to biodiversity powder showed decreased expression of CD69 activation marker upon stimulation and decreased expression of pro‐inflammatory cytokines IL‐1β, IL‐5, IL‐6, IL‐13, and TNF. These results encourage to develop biodiversity interventions for immunomodulation against immune‐mediated diseases.

## 5 MATERIALS AND METHODS

### 5.1 Plant and soil‐based powder

The plant and soil‐based powder used for the exposure was manufactured by the laboratory of environmental ecology, the University of Helsinki as described previously.^24^, ^28^ It comprised of a mixture of commercially available sieved composted materials made from crushed‐tree bark and mulch, dung, deciduous leaf litter, peat, and agricultural sludge, and commercially available dried and crushed Sphagnum moss (manufacturer: Biolan Oy, Eura, Finland). The composted ingredients were sieved and mixed thoroughly with the moss. The mixture was saturated with ultra‐pure mQ water, the mixture was hand‐squeezed in sterile conditions and filtered with a 250 µm sieve. The powder was collected in 50 ml glass tubes, frozen at −20°C, and freeze‐dried for 48 h. Finally, the material was inactivated using one autoclave round (30 min at 121°C) and the powder was kept at 4°C until use. The microbial contents of the powder were analyzed as triplicates before autoclaving using Illumina MiSeq. 16S rRNA gene metabarcoding with read length 2 × 300 bp using a v3 sequencing reagent kit, as in Hui et al.^57^ and Roslund et al.^58^, ^59^ (see Table S1). The raw sequences are publicly available in sequence read as BioProject ID (PRJNA753286).

### 5.2 Animals and ethical approval

A total of 16 female C57Bl/6J (Jax line 00664) mice from The Jackson Laboratory (Bar Harbor, ME), aged 7–8 weeks at the beginning of the exposure, were used in this study. Ethical approval for all animal work was provided by the Regional State Administrative Agency (AVI, animal permit number ESAVI/275/04.10.07/2018). Animals were housed in individual ventilated cages in the Tampere University animal facility. They were separated in groups of four animals per cage and exposed for 1 h to 50 ml inactivated powder, sprinkled on top of clean bedding in a new, clean cage. The dose was chosen based on visual verification that the mice became exposed to the powder. The powder was observed on furs, noses, and ears of the mice as well as a visible powder on cage bedding. The exposure was repeated on five consecutive days per week, for 3 weeks. Control animals were kept in a new cage with clean bedding for 1 h. The animals from different groups were fed with the same food and drinking water ad libitum but kept in different rooms and handled by different researchers to avoid cross‐contamination. At Day 21, mice were euthanized in CO_2_ chamber (Pre‐Set CO2 System‐1 Chamber, AS044AX, Able Scientific) and organs were subsequently collected for analysis.

### 5.3 Flow cytometric analysis

mLNs and spleen were collected from animals at Day 21. Single‐cell suspensions were prepared by mechanic dissociation of the organs by first mincing them and then straining them through a 40 µm cell strainer (Fisher Scientific) into PBS^−^^/^^−^ (pH 7.2, Thermo Fisher Scientific) supplemented with 1% heat‐inactivated FBS (Gibco, Thermo Fisher Scientific) and 2 mM ethylenediaminetetraacetic acid (EDTA). After centrifugation, red blood cells (RBCs) were lysed with 1 min ACK (Lonza) treatment and then washed with PBS containing 1% FBS and 2 mM EDTA. Cells were resuspended in PBS and stained with viability dye FVS510 (Becton Dickinson Biosciences) for 20 min, RT, followed by washing with FBS buffer. Fc receptors were blocked with Rat Anti‐Mouse CD16/CD32 antibodies (Mouse BD Fc Block™) for 5 min at 4°C before staining. Antibodies for flow cytometry were purchase from BD (CD3/FITC, CD4/PerCP‐Cy5.5, CD8a/APC‐H7, CD127/PE‐Cy7, CD25/Alexa Fluor 647, Foxp3/PE, CD44/APC, CD62L/BV786, CD69/V450). Staining was performed at 4°C for 20 min and cells were washed twice with fetal bovine serum (FBS) buffer before analyzing them. Foxp3 intracellular staining was performed after surface staining using Mouse Foxp3 Buffer set according to manufacturer instructions. CD4 was stained together with Foxp3 postpermeabilization. All samples were run with BD FACSAria Fusion and analyzed with FlowJo software (BD).

### 5.4 RNA extraction and RT‐qPCR analysis

RNA extraction was performed for spleen, lymph nodes, lung, small intestine, and colon samples. For spleen, small intestine, and colon TissueRuptor II was used according to manufacturer instructions (Qiagen). Briefly, 5–20 mg of tissue was collected in 2 ml Eppendorf tube containing 1 ml of TRIzol (Thermo Fisher Scientific). Using TissueRuptor II, the tissue was homogenized by five cycles of 40–50 Hz. Then, RNA extraction was conducted following RNeasy Mini Kit (Qiagen). Lung and lymph node samples were homogenized using ceramic beads with PowerLyzer® 24 homogenizer (3200 rpm, 45 s) and the RNA was extracted using RNeasy Plus Universal Mini Kit (all QIAGEN). Using iScript™ cDNA Synthesis Kit (Biorad) cDNA synthesis from RNA samples was performed. Gene expression was assessed using RT‐qPCR and used primers are listed in Table S4. The qPCR reaction was prepared using Luna® Universal qPCR Master Mix (New England Biolabs Inc.) and ABI QuantStudio 12 K Flex System (Thermo Fisher Scientific).

### 5.5 Serum cytokine analysis

Serums were collected on Day 21 after euthanasia of mice. Cytokines were measured using MILLIPLEX MAP Mouse TH17 Magnetic Bead Panel (MTH17MAG‐47K, Millipore Sigma) following manufacturer instructions. Fluorescence was analyzed with Bio‐Plex® 200 system (Bio‐Rad Laboratories) and data were collected with Bio‐Plex Manager software (Bio‐Rad Laboratories; v. 4.1). If cytokine concentrations were below detection limit for some serum samples, lowest detected concentration/2 (LOD/2) value of the specific cytokine was used for these samples in the statistical analysis.

### 5.6 T cell activation assay and supernatant analysis by MSD immunoassays

Spleen from animals was collected after euthanasia and single‐cell suspension was obtained as described in the previous section. After single‐cell suspension was obtained cells were rested overnight in RPMI 1640 with 10% FBS (Thermo Fisher Scientific), 1% l‐glutamine (Lonza), and 1% penicillin/streptomycin (Lonza). The next day splenocytes were stimulated with PMA 50 ng/ml (Thermo Fisher Scientific) and Ionomycin 1 ug/ml (Thermo Fisher Scientific) or left untreated as control. Cells were harvested at 4 h poststimulation and cells analyzed by flow cytometry as described above. Supernatants were collected at 24 h poststimulation and stored at −80°C for cytokine measurement using the Meso Scale Discovery U‐plex kit Biomarker Group 1 (Catalog K15069L‐1 and K15069L‐2), which include the following analytes: IFN‐ɣ, IL‐12p70, IL‐13, IL17A, IL‐17E/IL‐25, IL‐1β, IL‐5, IL‐6, TNF, IL‐17F, and IL‐33. Samples were also analyzed using MILLIPLEX MAP Mouse TH17 Magnetic Bead Panel (MTH17MAG‐47K, Millipore Sigma) as mentioned previously.

### 5.7 Gut bacterial community profiling

Total DNA from the stool samples was extracted using DNeasy PowerSoil Kit (QIAGEN GmbH). The V4 region of the bacterial 16S rRNA gene was amplified as described earlier.^60^ Negative controls (water) were used throughout the sequencing procedure. Paired‐end sequencing (2 × 250 bp) was performed using the MiSeq platform (Illumina).

The 16S rRNA gene read pairs were demultiplexed and reads were merged using USEARCH, version 10 with default parameters.^61^ Quality filtering was done using USEARCH fastq_filter command discarding reads containing above 1.0% expected errors. Operational taxonomic unit (OTUs) clustering with 97% similarity cutoff was performed using UPARSE algorithm,^62^ which also removes chimeric sequences. Taxonomies were determined using the RDP training set 16S rRNA database, version 16 with confidence threshold of 0.8. Low abundance (<5) OTUs and OTUs identified as mitochondrial, archaeal or chloroplast rRNA sequences were removed. Samples were rarefied to 1105 sequences per sample.

### 5.8 Statistical analysis

Normal distribution was assessed for each individual data set using Anderson–Darling tests or Shapiro–Wilk test when data sets did not fulfil the requirements (*α* = 0.05). Nonparametric data were analyzed using a Mann−Whitney *U* test (*α* = 0.05, two tailed), parametric data were analyzed using a two tailed *t* test, as appropriate (α = 0.05). All immunological data sets were analyzed using GraphPad Prism 8.0 (GraphPad Inc.). The statistical analyses for the gut microbiota were performed in R^63^ with package *phyloseq*.^64^ Shannon diversity index was used for analyzing alpha diversity, and community compositional differences were analyzed using Bray–Curtis dissimilarity metric and Permutational Multivariate Analysis of Covariance (PERMANOVA).

## 6 ADELE RESEARCH GROUP

Damiano Cerrone: Faculty of Built Environment, Tampere University, Tampere, Finland; Anna TH Luukkonen: Faculty of Built Environment, Tampere University, Tampere, Finland; Iida Mäkelä: Horticulture Technologies, Natural Resources Institute Finland, Turku, Finland; Hui Nan: Faculty of Built Environment, Tampere University, Tampere, Finland; Noora Nurminen: Faculty of Medicine and Health Technology, Tampere University, Tampere, Finland; Sami OIkarinen: Faculty of Medicine and Health Technology, Tampere University, Tampere, Finland; Anirudra Parajuli: Center for infectious medicine (CIM), Department of Medicine, Karolinska Institutet, Huddinge, Sweden;Mika Saarenpää: University of Helsinki, Helsinki, Finland; Yan Sun: University of Helsinki, Helsinki, Finland; Olli H Laitinen: Faculty of Medicine and Health Technology, Tampere University, Tampere, Finland; Marja I Roslund: University of Helsinki, Helsinki, Finland; Juho Rajaniemi: Faculty of Built Environment, Tampere University, Tampere, Finland; Heikki Hyöty: Faculty of Medicine and Health Technology, Tampere University, Tampere, Finland; Aki Sinkkonen: Horticulture Technologies, Natural Resources Institute Finland, Turku, Finland.

## CONFLICT OF INTERESTS

H. H., O. H. L., and A. S. are stakeholders and members of the board of Uute Scientific LtD., which develops biodiversity‐based interventions for the prevention of immune‐mediated disease. A. S., H. H. O. H. L., N. N., and S. O. have been named as inventors in a patent application “immunomodulatory compositions” submitted by the University of Helsinki (patent application number 20165932 at Finnish Patent and Registration Office). A. P., M. I. R., and A. S. have been named as inventors in a patent application “Immunomodulatory gardening and landscaping material” submitted by the University of Helsinki (patent application number 175196 at Finnish Patent and Registration Office). None of the inventors have received royalties from the patent applications.

## AUTHOR CONTRIBUTIONS

Martín Ignacio González‐Rodríguez, Noora Nurminen, Laura Kummola, Olli H. Laitinen, Sami Oikarinen, Anirudra Parajuli, Tanja Salomaa, Iida Mäkelä, Marja I. Roslund planned and performed the experiments, Aki Sinkkonen, Heikki Hyöty and Ilkka S. Junttila conceived the idea, planned the experiments, analyzed the data, wrote the MS, and oversaw the project.

## Supporting information

Supporting information.

## Data Availability

Bacterial sequencing data is available on Sequence Read Archive (SRA) by NCBI with BioProject ID: PRJNA753286.
