# Supplementary material for: Effect of inactivated nature‐derived microbial composition on mouse immune system
Source: Immun Inflamm Dis. 2021 Dec 6;10(3):e579. doi: 10.1002/iid3.579 (PMC8926502; doi:10.1002/iid3.579)
Supplement: Supplementary file 1 — Supporting information. [file IID3-10-e579-s001.docx]

Supplemental material for MS:

**Effect of inactivated nature-derived microbial composition on mouse immune system**
Martín Ignacio González-Rodríguez 1, Noora Nurminen 1*, Laura Kummola 1,2*, Olli H. Laitinen 1*, Sami Oikarinen 1, Anirudra Parajuli 3, Tanja Salomaa 1, Iida Mäkelä 5, Marja I. Roslund 4, Aki Sinkkonen 5, Heikki Hyöty 1,2, Ilkka S. Junttila 1,2# and the ADELE research group♫


1 Faculty of Medicine and Health Technology, Tampere University, 33014 Tampere, Finland
2 Fimlab Laboratories, 33520 Tampere, Finland

3Center for infectious medicine (CIM), Department of Medicine, Karolinska Institutet, Huddinge, Sweden

4 University of Helsinki, 00100 Helsinki, Helsinki, Finland

5 Horticulture Technologies, Natural Resources Institute Finland, 20520 Turku, Finland

♫ADELE research group: Damiano Cerrone6, Anna T.H. Luukkonen4, Iida Mäkelä5, Hui Nan1, Noora Nurminen1, Sami Oikarinen1, Anirudra Parajuli3, Mika Saarenpää4, Yan Sun4, Olli H. Laitinen1, Marja I. Roslund4, Juho Rajaniemi6, Heikki Hyöty1, Aki Sinkkonen5

*These authors contributed equally to this work. #Corresponding author

Supplementary Figure 1

***Supplementary figure 1. T cell phenotyping in spleen and mLN.*** Mice were either treated with soil (n=8) or control (n=8) as indicated in Figure 1. On day 21 single-cell suspension were prepared from spleen and mesenteric lymph nodes (mLNs) . Cells were analyzed using flow cytometry, open circles represent control mice and closed circles soil treated animals. Percentage of Naïve (CD44-/CD62L+), effector memory (CD44+/CD62L-) and central memory (CD44+/CD62L+) CD8+ T cell proportion in spleen (**A**) or mLN (**B**). The percentage of CD69 positive cells within CD4+ and CD8+ T cell are shown for spleen (**C** ) or mLN (**D**). Moreover, percentage of CD69 positive cells from Naïve CD4 and CD8 T cells are shown for spleen and mLN (**E**). Next, percentage of CD69 positive cells from EM CD4 and CD8 T cells are shown for spleen and mLN (**F**). Finally, percentage of CD69 positive cells from CM CD4 and CD8 T cells are shown for spleen and mLN (**G**). Values are shown as per cents with median and SEM is indicated, n=8 mice per group.

Supplementary figure 2.


***Supplementary figure 2. T cell activation assay and quantification***. Mice were either treated with soil (n=8) or control (n=8) as indicated in Figure 1. On day 21, single cell suspension from splenocytes was prepared, cells were rested overnight and then either left unstimulated or stimulated with PMA/Ionomycin for 4 hours and splenocytes harvested. (A) Gating strategy for CD8 cells, upper panel untreated, lower panel PMA/Ionomycin treated cells. (B) Per cent of CD69 positive cells from all CD8 positive cells after PMA/Ionomycin treatment. (C) Per cent of CD69 positive cells from all CD4 positive cells after PMA/Ionomycin treatment.

Supplementary figure 3.

***Supplementary figure 3. Cytokine quantification in splenocyte supernatants 24 hours post stimulation***. Mice were either treated with soil (n=8) or control (n=8) as indicated in Figure 1. On day 21, single cell suspension from splenocytes was prepared, cells were rested overnight and then either left unstimulated or stimulated with PMA/Ionomycin for 24 hours and supernatants were harvested. Expression level of (**A**) IL-12, (**B**) IL-17A, (**C**) IL-17F, (**D**) IL-25, (**E**) IL-33, (**F**) IFN-ɣ are indicated. N=8 mice per group.

Supplementary Table 1.

| **Genus level** | Average | SD |
| --- | --- | --- |
| Flavobacteriaceae_unclassified Total | 880.67 | 74.12 |
| SBR1031_ge Total | 865.00 | 206.69 |
| uncultured_ge Total | 851.00 | 6.68 |
| Devosia Total | 355.67 | 32.50 |
| Terrimonas Total | 298.33 | 20.29 |
| TRA3-20_ge Total | 290.67 | 36.63 |
| Algoriphagus Total | 259.67 | 63.12 |
| Pedobacter Total | 238.00 | 9.63 |
| Ferruginibacter Total | 202.00 | 12.83 |
| Lacunisphaera Total | 198.00 | 34.65 |
| Microbacteriaceae_unclassified Total | 192.67 | 8.96 |
| BIrii41_ge Total | 191.67 | 16.50 |
| Xanthomonadaceae_unclassified Total | 183.33 | 14.88 |
| Bacteria_unclassified Total | 174.33 | 11.56 |
| Pseudarthrobacter Total | 170.33 | 63.29 |
| Gammaproteobacteria_unclassified Total | 154.67 | 33.00 |
| Parcubacteria_unclassified Total | 149.67 | 7.72 |
| Flavobacterium Total | 143.00 | 3.74 |
| Absconditabacteriales__ge Total | 139.00 | 12.75 |
| A4b_ge Total | 122.33 | 13.12 |
| Candidatus_Kaiserbacteria_ge Total | 116.67 | 18.57 |
| Xanthobacteraceae_unclassified Total | 113.33 | 11.26 |
| Vicinamibacteraceae_ge Total | 112.33 | 19.01 |
| Arenimonas Total | 109.33 | 8.73 |
| Lutibacter Total | 105.33 | 20.29 |
| PAUC26f_ge Total | 101.67 | 20.76 |
| Fluviicola Total | 99.67 | 14.20 |
| Rhizobiales_unclassified Total | 98.33 | 6.02 |
| Bauldia Total | 96.33 | 11.84 |
| Reyranella Total | 91.33 | 17.61 |
| Haliangium Total | 90.33 | 3.77 |
| Candidatus_Falkowbacteria_ge Total | 88.67 | 9.10 |
| Pir4_lineage Total | 86.00 | 10.98 |
| Comamonadaceae_unclassified Total | 84.67 | 16.74 |
| Candidatus_Campbellbacteria_ge Total | 84.00 | 3.74 |
| Pseudolabrys Total | 83.33 | 3.86 |
| Parcubacteria_ge Total | 83.00 | 7.79 |
| Phenylobacterium Total | 81.67 | 15.28 |
| Flavisolibacter Total | 80.67 | 14.29 |
| S0134_terrestrial_group_ge Total | 80.67 | 10.21 |
| 67-14_ge Total | 79.33 | 9.53 |
| Cellvibrio Total | 78.67 | 7.93 |
| Caulobacteraceae_unclassified Total | 77.67 | 7.41 |
| KD4-96_ge Total | 77.33 | 3.40 |
| Sphingomonadaceae_unclassified Total | 77.33 | 3.86 |
| Demequina Total | 76.00 | 6.68 |
| NB1-j_ge Total | 75.33 | 14.61 |
| Dokdonella Total | 74.67 | 15.33 |
| Pseudomonas Total | 74.00 | 9.90 |
| Truepera Total | 73.00 | 10.20 |
| Legionella Total | 72.67 | 10.96 |
| Ellin6067 Total | 70.67 | 8.81 |
| Anaerolinea Total | 69.33 | 15.11 |
| Chryseolinea Total | 67.33 | 15.86 |
| Edaphobaculum Total | 66.67 | 10.34 |
| Bdellovibrio Total | 64.00 | 7.07 |
| JGI_0001001-H03 Total | 63.33 | 3.40 |
| Rhodanobacter Total | 60.67 | 5.79 |
| Opitutus Total | 59.33 | 4.92 |
| Pedosphaeraceae_ge Total | 58.67 | 9.39 |
| Xanthomonadales_unclassified Total | 58.00 | 4.55 |
| Hydrogenedensaceae_ge Total | 56.33 | 6.55 |
| Sphingomonas Total | 55.33 | 8.26 |
| Brevundimonas Total | 54.67 | 9.67 |
| Bryobacter Total | 54.67 | 3.68 |
| Kapabacteriales_ge Total | 53.67 | 9.18 |
| AKYH767_ge Total | 53.00 | 14.31 |
| 37-13_ge Total | 51.33 | 3.40 |
| NS9_marine_group_ge Total | 51.33 | 4.03 |
| vadinHA49_ge Total | 51.00 | 13.44 |
| Hydrogenophaga Total | 49.67 | 6.85 |
| cvE6_ge Total | 49.33 | 0.47 |
| Altererythrobacter Total | 49.00 | 2.45 |
| Chryseobacterium Total | 48.67 | 20.29 |
| Methylotenera Total | 48.33 | 2.36 |
| Anaerolineaceae_unclassified Total | 48.00 | 8.49 |
| Planococcaceae_unclassified Total | 47.00 | 10.03 |
| Gemmatimonas Total | 46.67 | 10.53 |
| SJA-28_ge Total | 46.33 | 6.80 |
| Nocardioides Total | 45.67 | 11.09 |
| Amb-16S-1323_ge Total | 45.00 | 0.82 |
| Alphaproteobacteria_unclassified Total | 44.33 | 2.36 |
| 0319-6G20_ge Total | 43.67 | 7.41 |
| Iamia Total | 43.67 | 5.79 |
| Massilia Total | 43.67 | 5.44 |
| JG30-KF-CM66_ge Total | 43.33 | 0.47 |
| OM27_clade Total | 43.00 | 7.12 |
| Pirellula Total | 43.00 | 8.83 |
| Caulobacter Total | 41.67 | 12.66 |
| SM2D12_ge Total | 41.00 | 9.42 |
| CCD24_ge Total | 40.67 | 3.30 |
| Luteimonas Total | 40.33 | 11.26 |
| NS11-12_marine_group_ge Total | 40.33 | 7.54 |
| Pirellulaceae_unclassified Total | 39.67 | 10.21 |
| Rhizobiaceae_unclassified Total | 39.67 | 6.24 |
| Hirschia Total | 39.33 | 2.05 |
| JG30-KF-CM45_ge Total | 38.67 | 10.14 |
| Methylocaldum Total | 38.00 | 10.68 |
| OM190_ge Total | 38.00 | 8.04 |
| KD3-93_ge Total | 37.67 | 2.05 |
| Luteolibacter Total | 36.33 | 4.99 |
| Adhaeribacter Total | 35.33 | 3.09 |
| Herpetosiphon Total | 35.33 | 6.94 |
| Sphingobacteriaceae_unclassified Total | 35.33 | 13.89 |
| BD1-7_clade Total | 35.00 | 10.03 |
| Bacteroidetes_vadinHA17_ge Total | 34.67 | 8.73 |
| OLB14_ge Total | 34.33 | 2.05 |
| WD2101_soil_group_ge Total | 34.00 | 5.66 |
| Rhodobacteraceae_unclassified Total | 33.00 | 4.08 |
| IMCC26256_ge Total | 32.67 | 6.55 |
| Longimicrobiaceae_ge Total | 32.67 | 4.11 |
| Novosphingobium Total | 32.67 | 13.10 |
| Pseudorhodoplanes Total | 32.00 | 6.38 |
| MND1 Total | 31.00 | 7.79 |
| Steroidobacter Total | 30.33 | 7.36 |
| Pseudoxanthomonas Total | 30.00 | 3.74 |
| Candidatus_Peribacteria_ge Total | 29.67 | 6.34 |
| Hyphomicrobiaceae_unclassified Total | 29.33 | 12.28 |
| Anaerolineae_unclassified Total | 28.67 | 13.42 |
| Gemmatimonadaceae_unclassified Total | 28.67 | 4.50 |
| Abditibacterium Total | 28.00 | 6.48 |
| S085_ge Total | 28.00 | 0.82 |
| Chitinophagaceae_unclassified Total | 27.67 | 4.03 |
| Candidatus_Nomurabacteria_ge Total | 27.00 | 8.04 |
| Nitrosospira Total | 27.00 | 1.63 |
| Paeniglutamicibacter Total | 27.00 | 11.05 |
| Peredibacter Total | 27.00 | 2.16 |
| BIyi10 Total | 26.33 | 4.99 |
| Kazania_ge Total | 26.33 | 2.49 |
| OLB13 Total | 26.00 | 8.04 |
| Chthoniobacter Total | 25.67 | 6.94 |
| possible_genus_04 Total | 25.67 | 2.62 |
| Blastocatellaceae_unclassified Total | 25.33 | 4.78 |
| Arenibacter Total | 25.00 | 6.16 |
| Vermiphilaceae_ge Total | 25.00 | 2.16 |
| Dinghuibacter Total | 24.33 | 4.03 |
| Gaiellales_unclassified Total | 23.33 | 9.03 |
| R7C24_ge Total | 23.00 | 6.38 |
| Flavitalea Total | 22.67 | 2.49 |
| env.OPS_17_ge Total | 22.33 | 4.19 |
| SB-5_ge Total | 22.00 | 2.45 |
| Balneolaceae_unclassified Total | 21.67 | 5.73 |
| Parachlamydiaceae_unclassified Total | 21.33 | 2.05 |
| Fimbriimonadaceae_ge Total | 21.00 | 6.38 |
| Opitutaceae_unclassified Total | 20.67 | 4.50 |
| Pedomicrobium Total | 20.33 | 7.93 |
| Sericytochromatia_ge Total | 20.00 | 3.56 |
| Micropepsaceae_unclassified Total | 19.67 | 3.30 |
| Aquicella Total | 19.33 | 3.30 |
| Candidatus_Moranbacteria_ge Total | 19.00 | 3.56 |
| Devosiaceae_unclassified Total | 19.00 | 4.24 |
| Microtrichales_unclassified Total | 19.00 | 4.08 |
| AKYG1722_ge Total | 18.67 | 1.25 |
| Chlamydiales_unclassified Total | 18.67 | 4.03 |
| Conexibacter Total | 18.67 | 4.50 |
| Aquimonas Total | 18.33 | 4.99 |
| Planctomicrobium Total | 18.33 | 4.50 |
| CK06-06-Mud-MAS4B-21 Total | 18.00 | 3.56 |
| Nitrosomonas Total | 18.00 | 4.55 |
| TK10_ge Total | 18.00 | 1.41 |
| Pseudohongiella Total | 17.67 | 3.30 |
| Rhodanobacteraceae_unclassified Total | 17.67 | 1.70 |
| Rhodopseudomonas Total | 17.33 | 3.40 |
| Terrimicrobium Total | 17.00 | 0.82 |
| JTB23_ge Total | 16.67 | 1.25 |
| Microgenomatia_ge Total | 16.67 | 4.03 |
| Mucilaginibacter Total | 16.67 | 0.94 |
| PLTA13_ge Total | 16.67 | 4.50 |
| Candidatus_Magasanikbacteria_ge Total | 16.33 | 4.19 |
| Vitellibacter Total | 16.33 | 1.25 |
| Janibacter Total | 16.00 | 1.41 |
| OLB12 Total | 16.00 | 0.82 |
| Paludibaculum Total | 16.00 | 1.63 |
| Acidibacter Total | 15.67 | 3.30 |
| Panacagrimonas Total | 15.33 | 2.62 |
| Polaromonas Total | 15.33 | 3.09 |
| Roseiflexaceae_unclassified Total | 15.33 | 5.25 |
| Blastopirellula Total | 14.67 | 5.19 |
| IS-44 Total | 14.67 | 2.62 |
| FCPU426_ge Total | 14.33 | 0.94 |
| Mycobacterium Total | 14.33 | 2.49 |
| Sumerlaea Total | 14.33 | 3.09 |
| Aminobacter Total | 14.00 | 3.74 |
| Pedosphaeraceae_unclassified Total | 14.00 | 3.56 |
| Polyangiales_unclassified Total | 14.00 | 4.55 |
| OLB8 Total | 13.67 | 2.87 |
| Oligoflexus Total | 13.67 | 1.89 |
| Polyangiaceae_unclassified Total | 13.33 | 1.25 |
| RBG-13-54-9_ge Total | 13.33 | 3.30 |
| Steroidobacteraceae_unclassified Total | 13.33 | 1.25 |
| Candidatus_Zambryskibacteria_ge Total | 13.00 | 2.16 |
| KF-JG30-C25_ge Total | 13.00 | 4.32 |
| Beijerinckiaceae_unclassified Total | 12.67 | 5.31 |
| SWB02 Total | 12.67 | 3.09 |
| 11-24_ge Total | 12.33 | 4.19 |
| Burkholderiales_unclassified Total | 12.33 | 2.87 |
| UTBCD1 Total | 12.33 | 3.09 |
| Caldilineaceae_unclassified Total | 12.00 | 6.98 |
| KF-JG30-B3_ge Total | 12.00 | 6.48 |
| Prosthecobacter Total | 12.00 | 4.32 |
| PB19_ge Total | 11.67 | 2.49 |
| A21b_ge Total | 11.33 | 0.47 |
| Candidatus_Adlerbacteria_ge Total | 11.33 | 0.94 |
| Micavibrionales_unclassified Total | 11.33 | 3.86 |
| Neochlamydia Total | 11.33 | 1.25 |
| SC-I-84_ge Total | 11.33 | 2.62 |
| Taeseokella Total | 11.33 | 4.71 |
| BD2-11_terrestrial_group_ge Total | 11.00 | 3.27 |
| Candidatus_Protochlamydia Total | 11.00 | 2.83 |
| MWH-CFBk5_ge Total | 11.00 | 2.94 |
| Oxalobacteraceae_unclassified Total | 11.00 | 3.56 |
| Dyadobacter Total | 10.33 | 1.70 |
| Ellin6055 Total | 10.33 | 4.50 |
| SH-PL14 Total | 10.33 | 3.86 |
| Sphingopyxis Total | 10.33 | 5.25 |
| Candidatus_Chisholmbacteria_ge Total | 10.00 | 2.94 |
| BD7-11_ge Total | 9.67 | 0.47 |
| Demequinaceae_unclassified Total | 9.67 | 2.87 |
| Desulfobacterota_unclassified Total | 9.67 | 2.05 |
| Burkholderia-Caballeronia-Paraburkholderia Total | 9.33 | 2.62 |
| Marmoricola Total | 9.33 | 5.25 |
| Methyloligellaceae_unclassified Total | 9.33 | 2.05 |
| Roseimicrobium Total | 9.33 | 0.47 |
| Saccharimonadales_ge Total | 9.33 | 0.94 |
| Subgroup_17_ge Total | 9.33 | 2.05 |
| Chloroflexi_unclassified Total | 9.00 | 2.16 |
| Flavihumibacter Total | 9.00 | 1.41 |
| Nannocystis Total | 9.00 | 2.16 |
| WS2_ge Total | 9.00 | 2.16 |
| Babeliales_unclassified Total | 8.67 | 2.87 |
| Dongia Total | 8.67 | 4.64 |
| Persicitalea Total | 8.67 | 3.30 |
| Rokubacteriales_ge Total | 8.67 | 2.05 |
| Unknown_Family_ge Total | 8.67 | 4.03 |
| Lysobacter Total | 8.33 | 3.77 |
| Nannocystaceae_unclassified Total | 8.33 | 3.68 |
| Allorhizobium-Neorhizobium-Pararhizobium-Rhizobium Total | 7.67 | 0.94 |
| Aureispira Total | 7.67 | 2.49 |
| Candidatus_Pacebacteria_ge Total | 7.67 | 1.89 |
| Hyphomicrobium Total | 7.67 | 2.36 |
| Ohtaekwangia Total | 7.67 | 1.70 |
| Sphingobacteriales_unclassified Total | 7.67 | 9.43 |
| Subgroup_10 Total | 7.67 | 2.36 |
| Vicinamibacteraceae_unclassified Total | 7.67 | 2.62 |
| Bacteriovoracaceae_unclassified Total | 7.33 | 3.09 |
| Bacteroidia_unclassified Total | 7.33 | 2.87 |
| KD3-10 Total | 7.33 | 2.36 |
| SJA-15_ge Total | 7.33 | 3.86 |
| Acidimicrobiia_unclassified Total | 7.00 | 2.16 |
| Gallionellaceae_unclassified Total | 7.00 | 1.41 |
| Planctomycetales_unclassified Total | 7.00 | 0.82 |
| Solimonadaceae_unclassified Total | 7.00 | 2.94 |
| Aeromicrobium Total | 6.67 | 3.68 |
| Methylophilaceae_unclassified Total | 6.67 | 2.87 |
| Micrococcales_unclassified Total | 6.67 | 2.87 |
| Parvibaculum Total | 6.67 | 5.44 |
| Phycicoccus Total | 6.67 | 1.70 |
| Thermomicrobiales_unclassified Total | 6.67 | 2.62 |
| Gaiella Total | 6.33 | 2.05 |
| Rhodovastum Total | 6.33 | 1.25 |
| A0839_ge Total | 6.00 | 1.63 |
| Bacteroidetes_VC2.1_Bac22_ge Total | 6.00 | 2.94 |
| Bosea Total | 6.00 | 2.94 |
| Candidatus_Collierbacteria_ge Total | 6.00 | 0.82 |
| Phaselicystis Total | 6.00 | 1.41 |
| Bacillales_unclassified Total | 5.67 | 1.89 |
| Chitinophaga Total | 5.67 | 1.25 |
| Luteitalea Total | 5.67 | 4.11 |
| OPB41_ge Total | 5.67 | 2.62 |
| RS25G Total | 5.67 | 1.70 |
| UBA12409_ge Total | 5.67 | 1.70 |
| Candidatus_Azambacteria_ge Total | 5.33 | 1.70 |
| Candidatus_Solibacter Total | 5.33 | 1.25 |
| Nordella Total | 5.33 | 2.62 |
| Phreatobacter Total | 5.33 | 1.25 |
| Phycisphaeraceae_unclassified Total | 5.33 | 0.47 |
| Planctopirus Total | 5.33 | 3.30 |
| Puia Total | 5.33 | 3.68 |
| Tahibacter Total | 5.33 | 1.70 |
| Afipia Total | 5.00 | 1.41 |
| Asticcacaulis Total | 5.00 | 2.83 |
| Candidatus_Caldatribacterium Total | 5.00 | 2.83 |
| Labrys Total | 5.00 | 2.83 |
| Myxococcota_unclassified Total | 5.00 | 1.41 |
| PeM15_ge Total | 5.00 | 1.41 |
| Rhodopirellula Total | 5.00 | 0.82 |
| Sphaerobacter Total | 5.00 | 2.83 |
| Sphingorhabdus Total | 5.00 | 0.82 |
| Acetobacteraceae_unclassified Total | 4.67 | 1.25 |
| CCM11a_ge Total | 4.67 | 0.47 |
| Gemmata Total | 4.67 | 1.25 |
| Lineage_IIb_ge Total | 4.67 | 2.05 |
| Patescibacteria_unclassified Total | 4.67 | 0.47 |
| Solirubrobacterales_unclassified Total | 4.67 | 0.94 |
| Sporichthya Total | 4.67 | 2.36 |
| Streptomyces Total | 4.67 | 1.25 |
| Vicinamibacterales_unclassified Total | 4.67 | 2.05 |
| Actinobacteria_unclassified Total | 4.33 | 0.47 |
| AKIW781_ge Total | 4.33 | 1.70 |
| Anaeromyxobacter Total | 4.33 | 1.25 |
| Bacteriovorax Total | 4.33 | 0.94 |
| Candidatus_Berkiella Total | 4.33 | 0.47 |
| Cytophaga Total | 4.33 | 2.62 |
| Gitt-GS-136_ge Total | 4.33 | 1.70 |
| LWQ8_ge Total | 4.33 | 0.94 |
| Micromonosporaceae_unclassified Total | 4.33 | 2.87 |
| PHOS-HE36_ge Total | 4.33 | 2.49 |
| Planktosalinus Total | 4.33 | 1.25 |
| Polyangia_unclassified Total | 4.33 | 1.25 |
| Qipengyuania Total | 4.33 | 3.30 |
| Rubinisphaeraceae_unclassified Total | 4.33 | 1.25 |
| SAR324_clade_ge Total | 4.33 | 1.89 |
| Stella Total | 4.33 | 1.70 |
| Angustibacter Total | 4.00 | 2.94 |
| Armatimonadales_ge Total | 4.00 | 0.82 |
| Bacillaceae_unclassified Total | 4.00 | 4.97 |
| Candidatus_Woesebacteria_ge Total | 4.00 | 1.41 |
| DEV007_ge Total | 4.00 | 0.82 |
| Hydrogenispora Total | 4.00 | 0.82 |
| Paenibacillus Total | 4.00 | 1.41 |
| Proteobacteria_unclassified Total | 4.00 | 2.83 |
| Pseudonocardia Total | 4.00 | 1.63 |
| SG8-4_ge Total | 4.00 | 1.63 |
| Subgroup_22_ge Total | 4.00 | 4.24 |
| Thermobispora Total | 4.00 | 0.00 |
| UBA6140 Total | 4.00 | 3.27 |
| WWE3_ge Total | 4.00 | 0.82 |
| A4b_unclassified Total | 3.67 | 1.25 |
| AKYG587 Total | 3.67 | 1.25 |
| Candidatus_Ovatusbacter Total | 3.67 | 3.09 |
| Chitinophagales_unclassified Total | 3.67 | 3.86 |
| CL500-29_marine_group Total | 3.67 | 0.94 |
| Cyanobacteria_unclassified Total | 3.67 | 0.47 |
| Dadabacteriales_ge Total | 3.67 | 1.25 |
| Micrococcaceae_unclassified Total | 3.67 | 2.05 |
| Noviherbaspirillum Total | 3.67 | 1.25 |
| Saccharimonadales_unclassified Total | 3.67 | 1.25 |
| SM1A07_ge Total | 3.67 | 2.49 |
| Subsaxibacter Total | 3.67 | 0.47 |
| Thermomonospora Total | 3.67 | 1.70 |
| Candidatus_Captivus Total | 3.33 | 1.25 |
| Ellin517 Total | 3.33 | 0.94 |
| Ga0077536_ge Total | 3.33 | 0.94 |
| MB-A2-108_ge Total | 3.33 | 0.47 |
| Omnitrophales_ge Total | 3.33 | 1.25 |
| Taibaiella Total | 3.33 | 2.49 |
| bac2nit3_ge Total | 3.00 | 2.16 |
| Candidatus_Xiphinematobacter Total | 3.00 | 2.16 |
| CPla-3_termite_group_ge Total | 3.00 | 1.41 |
| D05-2_ge Total | 3.00 | 2.94 |
| MBNT15_ge Total | 3.00 | 1.63 |
| Oligoflexales_unclassified Total | 3.00 | 0.82 |
| Pla1_lineage_ge Total | 3.00 | 2.16 |
| Rheinheimera Total | 3.00 | 2.16 |
| Roseomonas Total | 3.00 | 0.82 |
| Saprospiraceae_unclassified Total | 3.00 | 1.63 |
| SBR1031_unclassified Total | 3.00 | 0.82 |
| SM1A02 Total | 3.00 | 0.82 |
| Acidipila Total | 2.67 | 0.94 |
| Aneurinibacillus Total | 2.67 | 0.47 |
| Blfdi19_ge Total | 2.67 | 1.25 |
| Candidatus_Peregrinibacteria_ge Total | 2.67 | 1.70 |
| Cellulomonas Total | 2.67 | 1.25 |
| Emticicia Total | 2.67 | 0.94 |
| EPR3968-O8a-Bc78_ge Total | 2.67 | 1.70 |
| Ignavibacteriales_unclassified Total | 2.67 | 0.47 |
| LD29 Total | 2.67 | 1.25 |
| Microvirga Total | 2.67 | 2.49 |
| Oceanibaculum Total | 2.67 | 0.47 |
| Simkaniaceae_unclassified Total | 2.67 | 1.70 |
| Spirochaetaceae_unclassified Total | 2.67 | 0.47 |
| Taonella Total | 2.67 | 0.47 |
| Tychonema_CCAP_1459-11B Total | 2.67 | 1.70 |
| Acidobacteriaceae__unclassified Total | 2.33 | 1.25 |
| Acidobacteriae_unclassified Total | 2.33 | 1.89 |
| Acidothermus Total | 2.33 | 0.94 |
| Alcaligenaceae_unclassified Total | 2.33 | 1.25 |
| Burkholderiaceae_unclassified Total | 2.33 | 1.89 |
| Crocinitomicaceae_unclassified Total | 2.33 | 0.47 |
| Cyclobacteriaceae_unclassified Total | 2.33 | 1.25 |
| Firmicutes_unclassified Total | 2.33 | 1.25 |
| Gracilibacteria_ge Total | 2.33 | 0.94 |
| Granulicella Total | 2.33 | 0.94 |
| IMCC26207 Total | 2.33 | 0.94 |
| Lacibacter Total | 2.33 | 0.47 |
| Microtrichaceae_unclassified Total | 2.33 | 0.47 |
| mle1-27_ge Total | 2.33 | 0.47 |
| Myxococcales_unclassified Total | 2.33 | 1.25 |
| Paenibacillaceae_unclassified Total | 2.33 | 0.47 |
| Rickettsiales_unclassified Total | 2.33 | 0.94 |
| Stenotrophobacter Total | 2.33 | 1.70 |
| Syntrophorhabdus Total | 2.33 | 0.47 |
| Waddlia Total | 2.33 | 0.47 |
| WX65_ge Total | 2.33 | 0.94 |
| Acidobacteriales_unclassified Total | 2.00 | 1.41 |
| Brevibacillus Total | 2.00 | 1.41 |
| Caenimonas Total | 2.00 | 0.82 |
| Candidatus_Paracaedibacter Total | 2.00 | 0.82 |
| Clostridiaceae_unclassified Total | 2.00 | 1.41 |
| Clostridium_sensu_stricto_1 Total | 2.00 | 1.41 |
| Defluviicoccus Total | 2.00 | 1.41 |
| Imperialibacter Total | 2.00 | 1.41 |
| Isosphaeraceae_unclassified Total | 2.00 | 1.41 |
| Methylobacter Total | 2.00 | 2.16 |
| mle1-7 Total | 2.00 | 2.16 |
| mle1-8_ge Total | 2.00 | 0.82 |
| Prolixibacteraceae_unclassified Total | 2.00 | 0.82 |
| Rhodococcus Total | 2.00 | 0.82 |
| Schlesneria Total | 2.00 | 1.41 |
| Segetibacter Total | 2.00 | 0.82 |
| Singulisphaera Total | 2.00 | 1.63 |
| Subgroup_5_ge Total | 2.00 | 1.41 |
| Syntrophobacteraceae_unclassified Total | 2.00 | 0.00 |
| Tepidisphaera Total | 2.00 | 2.16 |
| Tepidisphaeraceae_ge Total | 2.00 | 0.82 |
| Thermoleophilia_unclassified Total | 2.00 | 1.41 |
| Thiobacillus Total | 2.00 | 1.41 |
| Babeliaceae_ge Total | 1.67 | 1.25 |
| Bradymonadales_ge Total | 1.67 | 0.94 |
| Chthoniobacterales_unclassified Total | 1.67 | 0.94 |
| Duganella Total | 1.67 | 2.36 |
| Escherichia-Shigella Total | 1.67 | 0.47 |
| Ferrovibrio Total | 1.67 | 0.47 |
| Fimbriiglobus Total | 1.67 | 1.25 |
| GWA2-38-13b_ge Total | 1.67 | 0.47 |
| Hungateiclostridiaceae_unclassified Total | 1.67 | 1.70 |
| Hymenobacter Total | 1.67 | 0.94 |
| Ignavibacterium Total | 1.67 | 0.47 |
| Inquilinus Total | 1.67 | 1.70 |
| JdFR-76 Total | 1.67 | 2.36 |
| Kaistia Total | 1.67 | 0.47 |
| Ketobacter Total | 1.67 | 0.47 |
| Latescibacterota_ge Total | 1.67 | 0.94 |
| Nitrospira Total | 1.67 | 1.25 |
| RCP2-54_ge Total | 1.67 | 1.70 |
| Sandaracinaceae_unclassified Total | 1.67 | 0.94 |
| Sandaracinus Total | 1.67 | 0.47 |
| Shinella Total | 1.67 | 0.47 |
| Solimonas Total | 1.67 | 1.25 |
| Sphingobium Total | 1.67 | 0.47 |
| Thermoactinomycetaceae_unclassified Total | 1.67 | 0.94 |
| Thermomicrobium Total | 1.67 | 0.47 |
| TM7a Total | 1.67 | 1.25 |
| Vampirovibrionaceae_ge Total | 1.67 | 1.70 |
| Vicingus Total | 1.67 | 1.25 |
| WPS-2_ge Total | 1.67 | 0.47 |
| Xanthomonadaceae_ge Total | 1.67 | 1.25 |
| Acidobacteriota_unclassified Total | 1.33 | 0.47 |
| Acinetobacter Total | 1.33 | 1.89 |
| Ahniella Total | 1.33 | 0.94 |
| Aminicenantales_ge Total | 1.33 | 0.47 |
| B1-7BS_ge Total | 1.33 | 1.25 |
| Bacilli_unclassified Total | 1.33 | 0.94 |
| Castellaniella Total | 1.33 | 0.47 |
| Cephaloticoccus Total | 1.33 | 0.47 |
| Chujaibacter Total | 1.33 | 1.25 |
| Clostridium_sensu_stricto_12 Total | 1.33 | 0.47 |
| Coxiella Total | 1.33 | 1.25 |
| Cyanobacteriia_unclassified Total | 1.33 | 1.25 |
| Desulfuromonadaceae_ge Total | 1.33 | 1.25 |
| DTU014_ge Total | 1.33 | 0.94 |
| Erythrobacter Total | 1.33 | 1.25 |
| Exiguobacterium Total | 1.33 | 0.47 |
| Frankiales_unclassified Total | 1.33 | 0.94 |
| Hydrogenedensaceae_unclassified Total | 1.33 | 1.25 |
| KCM-B-112 Total | 1.33 | 1.25 |
| Longivirga Total | 1.33 | 0.94 |
| Methylobacterium-Methylorubrum Total | 1.33 | 0.47 |
| Microscillaceae_unclassified Total | 1.33 | 0.94 |
| MSB-3C8_ge Total | 1.33 | 0.94 |
| Nannocystaceae_ge Total | 1.33 | 1.25 |
| Pajaroellobacter Total | 1.33 | 0.47 |
| Pelagicoccus Total | 1.33 | 0.94 |
| Porticoccaceae_unclassified Total | 1.33 | 1.25 |
| Pseudomonadaceae_unclassified Total | 1.33 | 1.25 |
| Pseudoxanthobacter Total | 1.33 | 1.25 |
| Pusillimonas Total | 1.33 | 1.25 |
| Rhizobiales_Incertae_Sedis_unclassified Total | 1.33 | 0.47 |
| Solirubrobacter Total | 1.33 | 1.89 |
| Spirosomaceae_unclassified Total | 1.33 | 1.25 |
| Sporocytophaga Total | 1.33 | 1.25 |
| Streptacidiphilus Total | 1.33 | 0.94 |
| Streptosporangiaceae_unclassified Total | 1.33 | 1.25 |
| Thermoflavimicrobium Total | 1.33 | 0.94 |
| Thermostaphylospora Total | 1.33 | 0.94 |
| Tumebacillus Total | 1.33 | 0.94 |
| UA11_ge Total | 1.33 | 0.94 |
| Verrucomicrobium Total | 1.33 | 0.94 |
| 01D2Z36_ge Total | 1.00 | 0.82 |
| 1174-901-12 Total | 1.00 | 1.41 |
| 211ds20_ge Total | 1.00 | 0.82 |
| Aestuariicella Total | 1.00 | 0.82 |
| Aquamicrobium Total | 1.00 | 1.41 |
| Aquisphaera Total | 1.00 | 0.82 |
| Babeliaceae_unclassified Total | 1.00 | 0.82 |
| Berkelbacteria_ge Total | 1.00 | 1.41 |
| Bernardetia Total | 1.00 | 0.82 |
| Caldicoprobacter Total | 1.00 | 0.82 |
| Candidatus_Chloroploca Total | 1.00 | 1.41 |
| Candidatus_Megaira Total | 1.00 | 0.82 |
| Candidatus_Omnitrophus Total | 1.00 | 0.82 |
| Clostridium_sensu_stricto_13 Total | 1.00 | 1.41 |
| Cohnella Total | 1.00 | 0.00 |
| DS-100_ge Total | 1.00 | 0.00 |
| Gelidibacter Total | 1.00 | 0.82 |
| Gemmataceae_unclassified Total | 1.00 | 0.82 |
| GIF3_ge Total | 1.00 | 1.41 |
| Gryllotalpicola Total | 1.00 | 0.82 |
| GWC2-45-44_ge Total | 1.00 | 0.82 |
| Haloactinopolyspora Total | 1.00 | 0.82 |
| Ilumatobacteraceae_unclassified Total | 1.00 | 0.82 |
| KI89A_clade_ge Total | 1.00 | 1.41 |
| Ktedonobacteraceae_unclassified Total | 1.00 | 0.82 |
| Lachnospiraceae_unclassified Total | 1.00 | 0.82 |
| Lacihabitans Total | 1.00 | 1.41 |
| Lautropia Total | 1.00 | 0.00 |
| Leucobacter Total | 1.00 | 0.00 |
| Limnobacter Total | 1.00 | 0.82 |
| Limnochordaceae_ge Total | 1.00 | 0.82 |
| Lineage_IIa_ge Total | 1.00 | 0.82 |
| Longispora Total | 1.00 | 0.00 |
| Luteibacter Total | 1.00 | 0.82 |
| Membranicola Total | 1.00 | 0.82 |
| Methylobacillus Total | 1.00 | 0.82 |
| Methylomonadaceae_unclassified Total | 1.00 | 0.82 |
| Methylophilus Total | 1.00 | 0.00 |
| MSBL9_unclassified Total | 1.00 | 0.82 |
| Myxococcaceae_unclassified Total | 1.00 | 1.41 |
| Occallatibacter Total | 1.00 | 0.82 |
| Oikopleura Total | 1.00 | 0.00 |
| Phycisphaerae_unclassified Total | 1.00 | 1.41 |
| Pseudogracilibacillus Total | 1.00 | 1.41 |
| Reichenbachiella Total | 1.00 | 0.82 |
| Syntrophomonas Total | 1.00 | 0.00 |
| Syntrophus Total | 1.00 | 1.41 |
| Thermomonas Total | 1.00 | 0.82 |
| Uliginosibacterium Total | 1.00 | 0.82 |
| WCHB1-81_ge Total | 1.00 | 0.00 |
| WD260_ge Total | 1.00 | 0.82 |
| WS1_ge Total | 1.00 | 0.82 |
| WS4_ge Total | 1.00 | 0.82 |
| Yersiniaceae_unclassified Total | 1.00 | 1.41 |
| Actinomarinales_unclassified Total | 0.67 | 0.47 |
| Aequorivita Total | 0.67 | 0.47 |
| Agarilytica Total | 0.67 | 0.94 |
| AKAU3564_sediment_group_ge Total | 0.67 | 0.94 |
| Alterococcus Total | 0.67 | 0.47 |
| Ammoniphilus Total | 0.67 | 0.47 |
| Arachidicoccus Total | 0.67 | 0.47 |
| Arsenicitalea Total | 0.67 | 0.47 |
| Aureimonas Total | 0.67 | 0.47 |
| Blastocatella Total | 0.67 | 0.47 |
| BSV26_ge Total | 0.67 | 0.94 |
| Candidatus_Spechtbacteria_ge Total | 0.67 | 0.47 |
| Candidatus_Uhrbacteria_ge Total | 0.67 | 0.47 |
| Candidatus_Vogelbacteria_ge Total | 0.67 | 0.47 |
| Candidatus_Yonathbacteria_ge Total | 0.67 | 0.47 |
| CCM19a_ge Total | 0.67 | 0.47 |
| Chelativorans Total | 0.67 | 0.47 |
| Christensenellaceae_R-7_group Total | 0.67 | 0.47 |
| Comamonas Total | 0.67 | 0.47 |
| Corynebacterium Total | 0.67 | 0.47 |
| Crocinitomix Total | 0.67 | 0.47 |
| DEV008 Total | 0.67 | 0.47 |
| Enterococcus Total | 0.67 | 0.47 |
| Fermentimonas Total | 0.67 | 0.94 |
| Frateuria Total | 0.67 | 0.47 |
| Gemmatimonadota_unclassified Total | 0.67 | 0.47 |
| Halocella Total | 0.67 | 0.94 |
| HN-HF0106 Total | 0.67 | 0.47 |
| Hungateiclostridiaceae_ge Total | 0.67 | 0.94 |
| Immundisolibacter Total | 0.67 | 0.47 |
| Intrasporangiaceae_unclassified Total | 0.67 | 0.47 |
| Larkinella Total | 0.67 | 0.94 |
| Leptolinea Total | 0.67 | 0.94 |
| Magnetospiraceae_unclassified Total | 0.67 | 0.47 |
| Magnetospirillaceae_unclassified Total | 0.67 | 0.94 |
| Methylocella Total | 0.67 | 0.47 |
| Methylococcaceae_unclassified Total | 0.67 | 0.47 |
| Methylomicrobium Total | 0.67 | 0.47 |
| Methylovirgula Total | 0.67 | 0.47 |
| Microbispora Total | 0.67 | 0.94 |
| Moraxellaceae_unclassified Total | 0.67 | 0.47 |
| N9D0_ge Total | 0.67 | 0.47 |
| Parapusillimonas Total | 0.67 | 0.94 |
| Parviterribacter Total | 0.67 | 0.47 |
| Phaeodactylibacter Total | 0.67 | 0.47 |
| Polycyclovorans Total | 0.67 | 0.94 |
| Rhizorhapis Total | 0.67 | 0.94 |
| Rhodocyclaceae_unclassified Total | 0.67 | 0.47 |
| Rhodoligotrophos Total | 0.67 | 0.47 |
| Rubritaleaceae_unclassified Total | 0.67 | 0.47 |
| Rubrivirga Total | 0.67 | 0.47 |
| Sorangium Total | 0.67 | 0.47 |
| Sphingobacterium Total | 0.67 | 0.47 |
| Spirochaeta Total | 0.67 | 0.47 |
| Spirochaeta_2 Total | 0.67 | 0.94 |
| Spirochaetota_ge Total | 0.67 | 0.94 |
| Sporolactobacillaceae_unclassified Total | 0.67 | 0.47 |
| Subgroup_2_ge Total | 0.67 | 0.94 |
| Subgroup_21_ge Total | 0.67 | 0.94 |
| Subgroup_7_ge Total | 0.67 | 0.47 |
| Symbiobacterium Total | 0.67 | 0.94 |
| Terracidiphilus Total | 0.67 | 0.47 |
| Thalassobaculum Total | 0.67 | 0.47 |
| Thermanaerothrix Total | 0.67 | 0.47 |
| Thermobacillus Total | 0.67 | 0.47 |
| Thermobifida Total | 0.67 | 0.94 |
| UKL13-1 Total | 0.67 | 0.47 |
| vadinBA26_ge Total | 0.67 | 0.94 |
| Vampirovibrio Total | 0.67 | 0.47 |
| Vampirovibrionaceae_unclassified Total | 0.67 | 0.94 |
| Verruc-01 Total | 0.67 | 0.94 |
| Vulgatibacter Total | 0.67 | 0.47 |
| WC3-116_ge Total | 0.67 | 0.47 |

***Supplementary table 1***. Genus level taxonomies of the soil and plant-based powder used in this study (sequences per sample). Mean and standard deviation (SD) are based on triplicate analysis of the powder sample.

Supplementary Table 2.

|  | **F** | **P** |
| --- | --- | --- |
| **Exposed vs. controls** |  |  |
| day 0 | 1.19 | 0.27 |
| **day 7** | **2.46** | **0.02** |
| **day 14** | **2.08** | **0.03** |
| day 21 | 1.39 | 0.17 |
| **Within exposed** |  |  |
| day 0 vs. day 7 | 1.39 | 0.17 |
| day 0 vs. day 14 | 1.56 | 0.13 |
| **day 0 vs. day 21** | **3.12** | **0.006** |
| **Within controls** |  |  |
| **day 0 vs. day 7** | **3.02** | **0.01** |
| **day 0 vs. day 14** | **2.71** | **0.01** |
| **day 0 vs. day 21** | **2.44** | **0.03** |
| day 7 vs. day 14 | 1.14 | 0.31 |
| **day 7 vs. day 21** | **2.47** | **0.03** |
| day 14 vs. day 21 | 1.24 | 0.19 |

***Supplementary table 2.* Bacterial community differences analyzed with PERMANOVA between exposed and control mice and within both groups.** Bray-Curtis distances used for analyses, F statistics and P values reported

Supplementary Table 3.

|  | **Mean Abundance (sd)** | |  |  |
| --- | --- | --- | --- | --- |
|  | **Exposed** | **Control** | **P value** | **adj. P*** |
| **Day 0 (Before)** |  |  |  |  |
| Actinobacteria | 74.4 (29.6) | 71.9 (23.8) | 0.87 | 0.87 |
| Bacteroidetes | 602.4 (33.1) | 542.7 (139.2) | 0.28 | 0.56 |
| Deferribacteres | 12.3 (26.6) | 4.4 (6.9) | 0.64 | 0.77 |
| Firmicutes | 356.9 (61.8) | 445.4 (129.3) | 0.15 | 0.46 |
| Proteobacteria | 13.1 (6.1) | 10.3 (5.4) | 0.38 | 0.57 |
| Verrucomicrobia | 38.4 (26.7) | 20.6 (28.7) | 0.15 | 0.46 |
| **Day 7** |  |  |  |  |
| Actinobacteria | 58.5 (32.3) | 102.5 (33.6) | 0.05 | 0.10 |
| Bacteroidetes | 579.5 (123.4) | 665.4 (86.9) | 0.19 | 0.23 |
| Deferribacteres | **10.8 (15.9)** | **1 (1.8)** | **0.002** | **0.01** |
| Firmicutes | 389.5 (134.4) | 285.5 (106.2) | 0.16 | 0.23 |
| Proteobacteria | **22.8 (8)** | **11.4 (3.6)** | **0.007** | **0.02** |
| Verrucomicrobia | 37.6 (29.1) | 32.4 (40.4) | 0.43 | 0.43 |
| **Day 14** |  |  |  |  |
| Actinobacteria | 46.8 (18.2) | 67 (35.2) | 0.34 | 0.41 |
| Bacteroidetes | 565 (86.6) | 646.3 (93.6) | 0.14 | 0.28 |
| Deferribacteres | 18.8 (20.8) | 6.6 (15.2) | 0.03 | 0.16 |
| Firmicutes | 394.8 (92.6) | 339.5 (67.3) | 0.34 | 0.41 |
| Proteobacteria | 16.8 (7.8) | 14.8 (4.9) | 0.80 | 0.80 |
| Verrucomicrobia | 55.5 (44.7) | 22.6 (24.3) | 0.14 | 0.28 |
| **Day 21 (After)** |  |  |  |  |
| Actinobacteria | 28.4 (15.2) | 47.6 (27) | 0.11 | 0.17 |
| Bacteroidetes | 567.5 (200.7) | 593.3 (121.7) | 0.60 | 0.72 |
| Deferribacteres | 32.3 (29.6) | 4.9 (4.1) | 0.05 | 0.15 |
| Firmicutes | 424.3 (186.8) | 420.6 (109.4) | 0.96 | 0.96 |
| Proteobacteria | 10.4 (5) | 16.6 (7.8) | 0.07 | 0.15 |
| Verrucomicrobia | 36.4 (19.4) | 16.6 (19.3) | 0.07 | 0.15 |
| **Within group comparisons** | |  | **P value** | **adj. P*** |
| Actinobacteria | Exposed day 0 vs. day 21 | | **0.001** | **0.01** |
| Actinobacteria | Control day 7 vs. day 21 | | **0.007** | **0.04** |
| Proteobacteria | Exposed day 7 vs. day 21 | | **0.004** | **0.02** |

***Supplementary table 3.* Differences in bacterial phyla abundances between exposed and control mice and within both groups during the experiment.** Average abundances of each phylum in the stool samples were compared using Wilcox signed-rank test and the p-values were adjusted for multiple comparisons using Benjamini-Hochberg method. All statistically significant comparisons within the groups are listed in the lower part.

* P value adjusted for multiple comparisons.

Supplementary Table 4.

| Name | Sequence 5' - 3' | product size | Tm |
| --- | --- | --- | --- |
| mFoxp3 (forward) | TCAGGAGCCCACCAGTACA | 76 bp | 64,5 |
| mFoxp3 (reverse) | TCTGAAGGCAGAGTCAGGAGA |  | 64,3 |
| mGATA3 (forward) | TTATCAAGCCCAAGCGAAG | 75 bp | 62,8 |
| mGATA3 (reverse) | TGGTGGTGGTCTGACAGTTC |  | 63,9 |
| mIFNg (forward) | GGAGGAACTGGCAAAAGGAT | 85 bp | 64,2 |
| mIFNg (reverse) | TTCAAGACTTCAAAGAGTCTGAGG |  | 62,7 |
| mIL-2 (forward) | GCTGTTGATGGACCTACAGGA | 114 bp | 64 |
| mIL-2 (reverse) | TTCAATTCTGTGGCCTGCTT |  | 64,6 |
| mIL-4 (forward) | GAGAGATCATCGGCATTTTGA | 122 bp | 64 |
| mIL-4 (reverse) | AGCCCTACAGACGAGCTCAC |  | 63,6 |
| mT-bet (forward) | TCAACCAGCACCAGACAGAG | 110 bp | 64,3 |
| mT-bet (reverse) | AAACATCCTGTAATGGCTTGTG |  | 62,6 |
| mRORgt (forward) | CACTGCCAGCTGTGTGCT | 96 bp | 64,3 |
| mRORgt (reverse) | TGCAAGGGATCACTTCAATTT |  | 63,3 |
| mIL-17A (forward) | CAGGGAGAGCTTCATCTGTGT | 94 bp | 62,7 |
| mIL-17A (reverse) | GCTGAGCTTTGAGGGATGAT |  | 63,3 |
| miNOS (forward) | GGGCAGTGGAGAGATTTTGC | 140 bp | 65.9 |
| miNOS (reverse) | CCAGAGGGGTAGGCTTGTCT |  | 64.4 |
| mArg-1 (forward) | AAGAATGGAAGAGTCAGTGTGG | 132 bp | 62,1 |
| mArg-1 (reverse) | GGGAGTGTTGATGTCAGTGTG |  | 62,6 |
| mIL-10 (forward) | GCCCAGAAATCAAGGAGCAT | 162 bp | 57 |
| mIL-10 (reverse) | TGTAGACACCTTGGTCTTGGAG |  | 60 |
| mIL-21 (forward) | GCCAGATCGCCTCCTGATTA | 128 bp | 59,32 |
| mIL-21 (reverse) | CATGCTCACAGTGCCCCTTT |  | 60,9 |
|  |  |  |  |
|  | |  |  |

***Supplementary table 4. Primers for RT-qPCR in mouse.*** Primer sequence for each pair of primers is shown together with their predicted amplicon size (base pairs based on BLAST prediction) and their respective melting point (Tm ). Tm were predicted using R. D. Blake, Scott G. Delcourt, Thermal stability of DNA, Nucleic Acids Research, Volume 26, Issue 14, 1 July 1998, Pages 3323–3332.
